# Time course decomposition of cell heterogeneity in TFEB signaling states reveals homeostatic mechanisms restricting the magnitude and duration of TFEB responses to mTOR activity modulation

**DOI:** 10.1186/s12885-016-2388-9

**Published:** 2016-06-07

**Authors:** Paula Andrea Marin Zapata, Carsten Jörn Beese, Anja Jünger, Giovanni Dalmasso, Nathan Ryan Brady, Anne Hamacher-Brady

**Affiliations:** Lysosomal Systems Biology, German Cancer Research Center (DKFZ) and BioQuant, University of Heidelberg, Heidelberg, Germany; Systems Biology of Cell Death Mechanisms, German Cancer Research Center (DKFZ) and BioQuant, University of Heidelberg, Heidelberg, Germany; Department of Surgery, Heidelberg University Hospital, Heidelberg, Germany; W. Harry Feinstone Department of Molecular Microbiology & Immunology, Johns Hopkins University Bloomberg School of Public Health, 615 N. Wolfe St., Baltimore, MD 21205 USA

**Keywords:** Transcription Factor EB (TFEB), Mammalian target of rapamycin (mTOR), Autophagy, Lysosomes, Proteasome, Systems biology, Subpopulation dynamics, Single cell, Multispectral imaging cytometry

## Abstract

**Background:**

TFEB (transcription factor EB) regulates metabolic homeostasis through its activation of lysosomal biogenesis following its nuclear translocation. TFEB activity is inhibited by mTOR phosphorylation, which signals its cytoplasmic retention. To date, the temporal relationship between alterations to mTOR activity states and changes in TFEB subcellular localization and concentration has not been sufficiently addressed.

**Methods:**

mTOR was activated by renewed addition of fully-supplemented medium, or inhibited by Torin1 or nutrient deprivation. Single-cell TFEB protein levels and subcellular localization in HeLa and MCF7 cells were measured over a time course of 15 hours by multispectral imaging cytometry. To extract single-cell level information on heterogeneous TFEB activity phenotypes, we developed a framework for identification of TFEB activity subpopulations. Through unsupervised clustering, cells were classified according to their TFEB nuclear concentration, which corresponded with downstream lysosomal responses.

**Results:**

Bulk population results revealed that mTOR negatively regulates TFEB protein levels, concomitantly to the regulation of TFEB localization. Subpopulation analysis revealed maximal sensitivity of HeLa cells to mTOR activity stimulation, leading to inactivation of 100 % of the cell population within 0.5 hours, which contrasted with a lower sensitivity in MCF7 cells. Conversely, mTOR inhibition increased the fully active subpopulation only fractionally, and full activation of 100 % of the population required co-inhibition of mTOR and the proteasome. Importantly, mTOR inhibition activated TFEB for a limited duration of 1.5 hours, and thereafter the cell population was progressively re-inactivated, with distinct kinetics for Torin1 and nutrient deprivation treatments.

**Conclusion:**

TFEB protein levels and subcellular localization are under control of a short-term rheostat, which is highly responsive to negative regulation by mTOR, but under conditions of mTOR inhibition, restricts TFEB activation in a manner dependent on the proteasome. We further identify a long-term, mTOR-independent homeostatic control negatively regulating TFEB upon prolonged mTOR inhibition. These findings are of relevance for developing strategies to target TFEB activity in disease treatment. Moreover, our quantitative approach to decipher phenotype heterogeneity in imaging datasets is of general interest, as shifts between subpopulations provide a quantitative description of single cell behaviour, indicating novel regulatory behaviors and revealing differences between cell types.

**Electronic supplementary material:**

The online version of this article (doi:10.1186/s12885-016-2388-9) contains supplementary material, which is available to authorized users.

## Background

Autophagy, a process of lysosomal degradation essential for cellular homeostasis, is transcriptionally regulated by Transcription Factor EB (TFEB) [1–3], which coordinates the expression of genes involved in lysosome biogenesis, autophagy and endocytosis [1, 2, 4]. Under normal growth conditions TFEB is transiently recruited to the lysosomes through its interaction with active RAG GTPases at the lysosomal membrane [5]. Active RAG GTPases also recruit the anabolic kinase complex mTOR, which phosphorylates TFEB at serine S211 to promote its dissociation from the lysosome and binding with 14-3-3 protein family members, which retain TFEB in the cytoplasm and inhibit its transcriptional activity [5–7]. Upon amino acid starvation, RAG GTPases are inactivated [8] resulting in the loss of lysosomal recruitment of TFEB and mTOR. Consequently, the cytoplasmic pool of TFEB becomes dephosphorylated, leading to the dissociation from 14-3-3 proteins and ultimately to nuclear accumulation of TFEB. Besides amino acid starvation, pharmacological inhibition of mTOR and lysosomal stresses result in TFEB dephosphorylation and nuclear accumulation [7, 9]. In the nucleus, TFEB activates the transcription of the CLEAR network (Coordinated Lysosomal Expression and Regulation), which is composed of at least 471 direct targets, including a battery of lysosomal and autophagy genes [1].

Abnormalities in autophagic processes can lead to neurodegenerative diseases and cancer [10]. Moreover, recent studies have identified TFEB and other family members as key players for metabolic reprogramming in pancreatic cancer [11, 12]. Thus, TFEB presents an attractive target for manipulating the cellular autophagic capacity in disease treatment. To date, studies on TFEB have primarily focused on the role of mTOR-mediated regulation of nuclear-cytoplasmic TFEB shuttling. Intriguingly, transcription of TFEB-controlled autophagosomal and lysosomal genes is increased in cells overexpressing TFEB [1, 2, 6] and overall cellular TFEB protein levels are reduced following TFEB activation via long-term (15 hours) chloroquine-induced lysosomal stress [7], suggesting TFEB concentration changes may contribute to the regulation of TFEB signaling. However, the relationship between mTOR activity states and temporal changes in TFEB subcellular localization and concentration has not been elucidated. To that end, we performed time course analysis over 15 hours of TFEB levels and localization by quantitative Western blotting and imaging cytometry. We activated mTOR by fresh addition of fully-supplemented medium (FM), or inhibited mTOR by Torin1 treatment [13] or nutrient deprivation [14]. We report that overall cellular TFEB levels transiently decrease in response to small increases in mTOR activation, and transiently increase in response to mTOR inhibition. Both Western blot and population-averaged imaging results displayed high variability, suggesting that heterogeneous TFEB responses within the cell population may cache important information on these complex dynamics. We therefore analyzed single-cell imaging cytometry data using spanning-tree progression analysis of density-normalized events (SPADE) agglomerative clustering [15], as a basis for unbiased and quantitative detection of spatial and temporal dynamics of subpopulations. Using unsupervised clustering, we identified three TFEB phenotype subpopulations, with low, medium and high nuclear TFEB concentrations. We found that total cellular TFEB levels and subcellular localization are directly under control of a short-term rheostat controlled by mTOR. mTOR inhibition rapidly activates TFEB in a fraction of cells, for a limited duration, with distinct TFEB subpopulation re-inactivation dynamics in response to Torin1 *vs.* nutrient deprivation. Moreover, time course subpopulation analysis identified a correlation between TFEB protein levels and nuclear localization, and revealed differences between HeLa and MCF7 cells in the sensitivity of TFEB to mTOR regulation. Finally, subpopulation analysis revealed that in response to mTOR inhibition, maximal nuclear localization of TFEB is negatively regulated by the proteasome, independently of TFEB concentration.

## Methods

### Materials

Cell culture reagents were obtained from Invitrogen, Sigma, Lonza and PAN Biotech. Methanol-free paraformaldehyde was obtained from Alfa Aesar. Torin1 was purchased from Merck, DMSO from Genaxxon Biosciences and U0126 was from Biovision. Hoechst 33342 was purchased from ImmunoChemistry.

### Cell culture and treatments

The human cervical cancer cell line HeLa Kyoto and the human breast cancer cell line MCF7 (obtained from CLS Cell lines service, Heidelberg) were cultured in DMEM (1 g/L D-glucose, 0.11 g/L sodium pyruvate), supplemented with 2 mM L-Glutamine, 10 % Fetal Bovine Serum, non-essential amino acids and penicillin/streptomycin/amphotericin B. Cells were routinely tested for mycoplasma contamination using Hoechst 33342. Transient transfections were performed using jetPRIME (Polyplus) according to the manufacturer’s instructions. Transfection complexes were removed after 6 hours and experiments performed at 24 hours of expression. Nutrient deprivation (ND) was introduced using glucose-containing HBSS (Life Technologies; no. 14025), supplemented with penicillin/streptomycin/amphotericin B. For drug treatments, cells were incubated in FM or HBSS, containing one or a combination of the following reagents: Torin1 (2 μM), U0126 (10 μM), epoxomicin (1 μM), and actinomycin D (1 μg/ml). Co-treatments with epoxomicin, actinomycin D or DMSO included a pre-treatment period (Fig. 7c-e). Cells were pretreated with Epox, ActD or vehicle control (DMSO) for 1 hour, and subsequently treated with FM supplemented with Torin1 in combination with the respective pretreatment reagent for 1 hour. For pre-treatments the drugs were directly added to the culture medium, without addition of fresh FM.

### Cloning

Entry Clones were obtained from the German cDNA Consortium of the German Cancer Research Center. N-terminally tagRFP-tagged clone of 14-3-3 protein isoform YWHAG, RFP-YWHAG, was generated using the Gateway Cloning System (Life Technologies). TFEB wild type was cloned using forward primer: 5′-gtaAAGCTTcgatggcgtcacgcatagggttgcgcatg-3′ and reverse primer 5′- tacGGTACCttacagcacatcgccctcctccat-3′ and inserted into pEGFP (Invitrogen) generating TFEB with N-terminal GFP fusion, GFP-TFEB.

### Immunofluorescence and fluorescence microscopy

Fifty thousand cells were plated per well of an 8 well μ-slide microscopy chamber (ibidi) 24 hours before treatment. Following drug treatments, cells were fixed with 4 % paraformaldehyde in PBS for 15 minutes, permeabilized with 0.3 % Triton X-100 in PBS for 10 minutes and blocked with 3 % BSA in 0.3 % Triton X-100/PBS for 1 hour. Cells were then incubated with primary antibodies against LAMP1 (Hybridoma Bank; #H4A3-s), TFEB (Cell Signaling; #4240S), or p-4E-BP1 (Cell Signaling; #2855S) in 0.3 % Triton X-100/PBS at 4 °C overnight. Fluorescence staining was performed using anti-rabbit Alexa Fluor 488 or 594 secondary antibodies (Life Technologies; #A11008, #A11012) in 0.3 % Triton X-100/PBS at room temperature for 1 hour. Fluorescence microscopy was performed with a DeltaVision microscope system (Applied Precision) using a 60x oil immersion objective (Olympus) and a digital CCD camera (Hamamatsu Photonics). Following acquisition, images were deconvolved with Softworks V3.5.1 (Applied Precision) to increase spatial resolution. Images were prepared using ImageJ (rsbweb.nih.gov/ij/). Representative images shown are total intensity projections (Z-axis scans).

### Immunoblotting

Six hundred thousand cells/well were plated in 6-well plates, 24 hours prior drug treatment. Following drug treatments whole cell lysates were prepared of adherent and floating cells with RIPA lysis buffer containing 1X EDTA-free protease inhibitor cocktail (Roche) and 2X PhosphoSTOP (Roche). Dosed protein samples were separated on pre-cast 4–12 % Bis-Tris gels (Invitrogen) and transferred to nitrocellulose using the iBlot dry blotting system (Invitrogen). Blocked membranes were incubated with primary antibodies against TFEB (#101532; Santa Cruz), LAMP1 (# H4A3-s; Hybridoma Bank), LC3 (#2775; Cell Signaling), 4E-BP1 (#9452; Cell Signaling), phospho-4E-BP1 (#9459S; Cell Signaling), p70-S6K1 rabbit IgG (#9202S; Cell Signaling), phospho-p70-S6K1 (#9205S; Cell Signaling) and GAPDH (#25778; Santa Cruz). HRP-conjugated anti-rabbit IgG (#213110-01; GeneTex) and anti-mouse IgG (#213111-01; GeneTex) antibodies were used as secondary antibodies. For immunodetection membranes were incubated with peroxide and luminol solution (1:1) and analyzed with a chemiluminescence imager (Intas). Protein bands were quantified using the gel analysis tool of ImageJ and normalized to the loading control GAPDH. Blots shown are representative of at least three independent experiments.

### Multispectral imaging cytometry

Flow cytometry coupled to high resolution imaging was performed using the ImageStreamX cytometer operated with INSPIRE 4.1.501.0 software (Amnis), using a 40X air objective.

#### Data collection

Two hundred fifty thousand cells per well of a 12-well plate were plated on the day before drug treatments. Following drug treatments, cells were trypsinized, harvested by centrifugation at 800 g for 5 minutes at 4 °C and fixed with 4 % paraformaldehyde for 15 minutes at room temperature. For detection of endogenous TFEB, cells were immunostained as stated above. Nuclei were labeled with 1 μg/mL Hoechst 33342 in PBS for 10 minutes. Compensation controls were generated from single-color control cells. Endogenous TFEB was immunostained with an antibody against TFEB and Alexa Fluor 594. Lysosomes were immunostained with an antibody against LAMP1 and Alexa Fluor 647. For measurements cells were resuspended in PBS. Fluorescence signal of Hoechst 33342 was excited using the 405 nm laser and detected in channel 1 (420–480 nm). Alexa Fluor 594 was excited using the 561 nm laser and the fluorescence signal was detected in channel 4 (595–642 nm).

#### Data processing

All data processing was performed using the IDEAS v6.0 software (Amnis). For each treatment and time point a total of 10000 cells were collected. Following compensation, cells were gated as single (based on the area and aspect ratio of the bright filed mask) and in-focus (based on the Gradient RMS of the bright filed image). With the exception of 15 hours, at least 2000 cells were analyzed after gating. The nuclear, cytoplasmic and cellular masks of gated cells were calculated based on the following morphological and logical operations: Cell, default mask for TFEB channel *OR* 5-pixel erosion of default bright field mask; Nucleus, 70 % Threshold mask on Hoechst channel; Cytoplasm: Cell *AND NOT* Nucleus. The features “Intensity Cell, Nucleus and Cytoplasm” were calculated as the total intensities (background subtracted) in their respective masks. The features “Concentration Cell, Nucleus and Cytoplasm” were calculated by dividing the intensity features by the area of their respective masks (in *μm*^2^). The feature “Nuclear percentage” was calculated as the ratio between the features “Intensity Nucleus” and “Intensity Cell”, multiplied by 100. The feature “Max Contour Position” was calculated with a build in function available in IDEAS software [16]. The feature “Mean Pixel Nu/Cyto” was calculated based on masks which underestimated the nuclear and cytoplasmic compartments in order to avoid including cytoplasmic pixels in the nuclear signal or including background or nuclear pixels in the cytoplasmic signal. Underestimated masks were obtained by morphological erosion of the original masks. These feature values were exported to.fcs-files for further processing with the clustering software SPADE V2.0 (Spanning-tree Progression Analysis of Density-normalized Events) [15]. The number of clusters and combination of input features was optimized as presented on the Results Section. The remaining SPADE input parameters were set to default values (arcsinh with cofactor = 5, neighborhood size = 5, local density approximation factor = 1.5, max allowable cells in pooled down-sampled data = 50000, fixed number of cells remained = 20000, Algorithm: K-means). Clustering results were exported to.fcs files and subsequently converted to .txt files using the IDEAS software. Text files were imported into MATLAB R2014a for data representation and further analysis.

#### Data representation

Mean population responses were obtained by averaging the single-cell data from a specific treatment, time point and repetition. All subpopulations were identified according to the classification model obtained from FM and Torin1 data in Fig. 4 (Refer to Additional file 1: Figure S1 for further explanation of the classification work flow).

### Statistical comparisons

Statistical comparisons were performed with Student’s two-tailed t-test or the non-parametric test Wilcoxon-rank-sum (two-sided). The latter was used in inter-cluster comparisons of non-normally distributed variables, including the features “Mean Pixel Nuc/Cyto” (Figs. 4b, d and 5b) and the discrete variable “LAMP1 Max Contour Position” (Fig. 6d).

## Results

### Regulation of TFEB localization and protein levels by mTOR

**Fig. 1:**
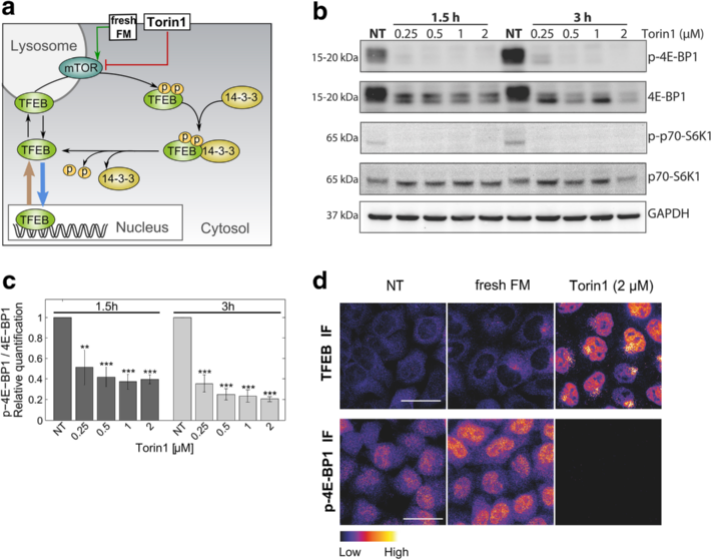
Characterization of the effect of Torin1 and fresh nutrients on mTOR and endogenous TFEB. **a** Schematic representation of the effects of Torin1 and fresh fully-supplemented medium (FM) on the regulation of TFEB by mTOR. **b** Dose-response of the effect of Torin1 on mTOR activity. HeLa cells were treated with FM containing the indicated concentrations of Torin1, or kept in culture medium (non-treated, NT), for 1.5 or 3 hours, and phosphorylation of the mTOR substrates 4E-BP1 and p70-S6K1 was measured by Western blotting. **c** Quantification of the ratio of phosphorylated 4E-BP1 (p-4E-BP1) to total 4E-BP1. Graphs display mean values of three independent experiments normalized to NT values. Error bars denote mean ± SD of three independent experiments. Statistical significance was tested vs. NT conditions (Student’s two-tailed t-test; **, *p* ≤ 0.01; ***, *p* ≤ 0.001). **d** Immunofluorescence of TFEB and p-4E-BP1, as a read-out for mTOR activity, in response to FM and Torin1. HeLa cells were kept in culture medium (non-treated, NT), treated with fresh FM, or with FM supplemented with Torin1 (2 μM) for 3 hours and immunostained for TFEB and p-4E-BP1. To reveal varying intensity levels the look-up-table ‘Fire’ (ImageJ) was applied to grey scale images, representing intensity values ranging from low (dark purple) to high (white) as displayed in color scale bar. Scale bars, 20 μm

We first established conditions for suppressing mTOR activity with the specific inhibitor Torin1 [13], and increasing mTOR activity by the renewed addition of fully-supplemented medium (FM) (illustrated in Fig. 1a). Torin1-mediated TFEB activation has been reported for concentrations ranging from 0.25 μM [5, 6, 9] to 2 μM [7]. Thus, we treated HeLa cells with 0.25 to 2 μM Torin1 for 1.5 and 3.0 hours, and determined the phosphorylation state of the mTOR substrates 4E-BP1 and p70-S6K1 by Western blot. Maximal inhibition of 4E-BP1 and p70-S6K1 phosphorylation was achieved in response to 2 μM after 3 hour treatment (Fig. 1b, c). High-resolution imaging further demonstrated that at 3 hours of treatment with 2 μM Torin1, immunofluorescence detection of phosphorylated 4E-BP1 was fully suppressed, and nuclear accumulation of TFEB was potently induced (Fig. 1d). Interestingly, total cellular TFEB immunofluorescence appeared strongly increased under Torin1 treatment, suggesting that mTOR inhibition increased TFEB protein levels. Of note, the addition of fresh FM resulted in an increased immunofluorescence signal of phosphorylated 4E-BP1, indicating mTOR activation by the replenished metabolic substrates and growth factors present in fresh FM. Consistent with increased mTOR activity, TFEB was predominantly retained in the cytoplasm under fresh FM conditions.

### Time course quantification of TFEB response to modulations in mTOR activity by Western blot analysis

**Fig. 2:**
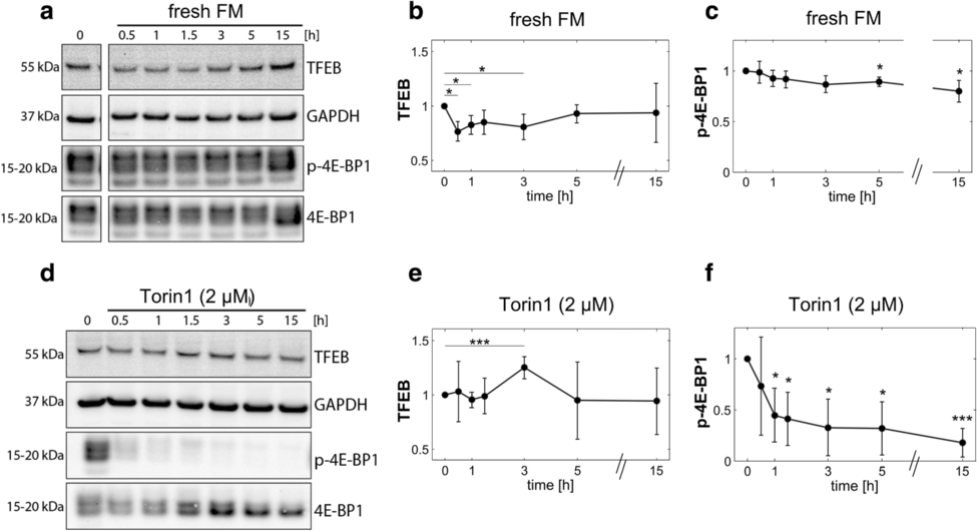
Quantitative Western blot analysis of TFEB protein levels in response to mTOR activity modulations. **a** HeLa cells were treated with fresh FM to enhance mTOR activity. At the indicated time points, levels of TFEB and phosphorylated 4E-BP1 (p-4E-BP1) were analyzed by Western blotting. Lanes for time point ‘0’ originate from same membrane as later time points. **b** Quantified values for TFEB, normalized to loading control GAPDH, shown relative to time point ‘0’. **c** Quantified values for p-4E-BP1, normalized to total 4E-BP1, shown relative to time point ‘0’. **d** HeLa cells were treated with FM containing 2 μM Torin1. At the indicated time points, levels of TFEB and phosphorylated 4E-BP1 (p-4E-BP1) were analyzed by Western blotting. **e** Quantified values for TFEB, normalized to loading control GAPDH, shown relative to time point ‘0’. **f** Quantified values for p-4E-BP1, normalized to total 4E-BP1, shown relative to time point ‘0’. Error bars denote mean ± SD of three independent experiments. Statistical significances were tested vs. time point ‘0’ (Student’s two-tailed t-test; *, *p* ≤ 0.05; ***, *p* ≤ 0.001)

To quantitatively investigate the effect of mTOR activity modulation on TFEB protein levels we treated HeLa cells with either fresh FM alone or containing 2 μM Torin1 over a time course of 15 hours, and measured levels of TFEB and phosphorylated 4E-BP1 by quantitative Western blot. Following the addition of fresh FM, TFEB protein levels were significantly decreased between 0.5, 1 and 3 hours, followed by a prolonged recovery to basal levels (Fig. 2a, b). In parallel, 4E-BP1 phosphorylation was stable in the first hour and then decreased over time, significantly at 5 and 15 hours (Fig. 2a, c). Notably, in contrast to imaging results (Fig. 1d), a significant increase in 4E-BP1 phosphorylation at 3 hours was not detected after addition of FM (Fig. 2c), indicating that in response to the addition of fresh FM mTOR is only mildly up-regulated, at levels below the sensitivity of Western blot analysis. However, at time points of 5 and 15 hours the reduction to 4E-BP1 phosphorylation suggests a progressive and significant reduction to mTOR activity.

Torin1 (2 μM) treatment on the other hand initially resulted in stable TFEB protein levels, which increased significantly after 3 hours (Fig. 2d, e), similar to as observed by imaging (Fig. 1d). Similar to following long-term (15 hour) mTOR inhibition with chloroquine [7], following 5 and 15 hours of Torin1 treatment TFEB levels were reduced (Fig. 2d, e), albeit with a high degree of variation between experiments. Importantly, Torin1 inhibition of 4E-BP1 phosphorylation was maintained also at 15 hours (Fig. 2f). These findings suggest that total TFEB levels are oppositely regulated by Torin1 and FM treatments during the initial 3-hour treatment period, followed by a prolonged TFEB recovery to initial levels. However, for most time points, the variability of immunoblotting data was too high to infer dynamic behavior of TFEB.

### Mean population time course quantification of TFEB response to modulations in mTOR activity by multispectral imaging cytometry

#### Multispectral imaging cytometry for quantification of endogenous TFEB in cell populations

TFEB exerts its activity in the nucleus, and thus spatial dynamics of TFEB signaling contain relevant information. We therefore performed single-cell analysis of TFEB subcellular localization and protein levels in cell populations using the imaging cytometer, ImageStreamX (ISX) [17]. For each obtained single-cell image, the nuclear and cytoplasmic compartments were segmented, and based on these masks and the total fluorescence intensity of endogenous TFEB, two normalized features were calculated to report spatial concentration states. The first feature, “Mean Pixel Nuc/Cyto”, reflects the nuclear/cytoplasmic ratio of TFEB concentration, and was calculated as the ratio of the mean pixels from each compartment. The second feature, referred to as “Concentration”, was determined by normalizing the total intensity of TFEB to the cell area (described in Methods).

**Fig. 3:**
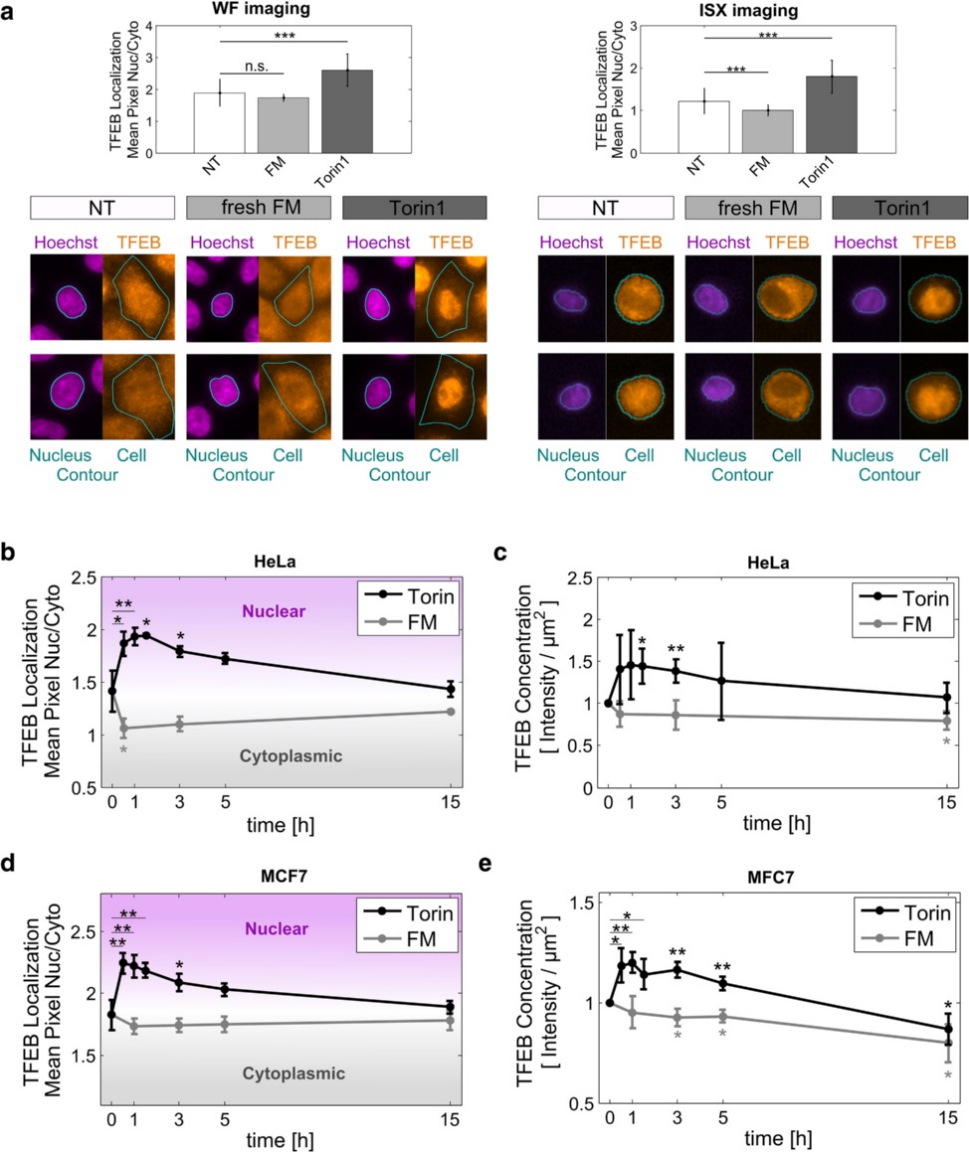
Multispectral imaging cytometry quantification of TFEB localization and levels in response to mTOR activity modulations. Cells were kept in culture medium (NT, non-treated), or treated with fresh FM or FM supplemented with Torin1 (2 μM). Following, cells were immunostained for TFEB and nuclei labelled with Hoechst 33342. **a** Representative fluorescence images and quantified TFEB nuclear localization in HeLa cells at 3 hours of treatment, measured with high-resolution wide field imaging (WF, *left panels*) or with the multispectral imaging cytometer ImageStreamX (ISX, *right panels*). Graphed values represent the mean ± SD nuclear/cytoplasmic ratio of 25 to 30 randomly selected cells of one representative experiment from three independent repetitions. Statistical significances were tested vs. NT control (Student’s two-tailed t-test; ***, *p* ≤ 0.001; n.s., *p* > 0.05). **b**-**e** Time course of mean population response of TFEB subcellular localization and protein levels for treatments with Torin1 or fresh FM, in HeLa and MCF7 cell lines. Concentrations are shown relative to time point ‘0’. Reported values represent the mean among three independent experiments ± SD. Statistical significances were tested vs. time point ‘0’, which corresponds to the NT control (Student’s two-tailed t-test; *, *p* ≤ 0.05; **, *p* ≤ 0.01)

To gauge the sensitivity of imaging cytometry (ISX) for assessing TFEB subcellular localization we compared the nuclear/cytoplasmic ratios of TFEB (Fig. 3a), and TFEB and Hoechst intensity profiles (Additional file 2: Figure S2), between ISX and high-resolution wide field imaging (WF) data-sets. Extended sets of representative ISX images for each condition are presented in Additional file 3: Figure S3. Similar intensity profiles were obtained with both techniques for all conditions. Furthermore, the nuclear/cytoplasmic ratios obtained for WF were higher than for ISX measurements. However, qualitatively similar FM and Torin1 responses were obtained. Both imaging approaches reported reduced nuclear localization in response to 3 hour FM treatment (8 % reduction for WF and 19 % for ISX) and increased nuclear localization upon treatment with Torin1 (38 % increase for WF and 47 % for ISX). We thus conclude that the ISX approach is more sensitive. Moreover, higher population sampling permits more robust quantitative analysis of relative changes induced by conditions, and, importantly, allows for improved significance testing.

#### Time course quantification of mean TFEB responses to FM and Torin1 treatments

Next, we assessed the mean population responses in HeLa cells treated under the conditions and time points reported in Fig. 2. Initially (*t* = 0), TFEB displayed a slightly higher concentration in the nuclear compartment, with a nuclear/cytoplasmic ratio of 1.4 (Fig. 3b). Upon treatment with fresh FM, at 0.5 hours the nuclear/cytoplasmic ratio rapidly decreased (from 1.4 to 1.0), and then gradually increased back to initial levels during the later time points. Consistent with Western blot findings, fresh FM induced a rapid, 14 % decrease in mean total cell TFEB concentrations within 0.5 hours, which was maintained up to 15 hours (Fig. 3c).

Upon treatment with Torin1, at 0.5 hours the nuclear/cytoplasmic ratio increased (from 1.4 to 1.9), peaking at 1 hour, and following 1.5 hours was gradually reduced to a final distribution of 1.4 at 15 hours, similar to time point 0 (Fig. 3b). Consistent with Western blot findings, Torin1 increased cellular TFEB concentration (Fig. 3c), significantly at 1.5 (45 %) and 3 hours (38 %), after which concentrations were reduced to approximately initial (*t* = 0) levels.

We further evaluated the effect of fresh FM and Torin1 treatments in MCF7 cells. Consistent with the findings in HeLa cells, within 1 hour of treatment with fresh FM the TFEB nuclear/cytoplasmic ratio slightly but non-significantly decreased from 1.8 to 1.7 (Fig. 3d) and overall TFEB protein levels were reduced by 5 % (Fig. 3e). Conversely, Torin1 treatment increased the nuclear/cytoplasmic ratio to 2.2 (Fig. 3d) and increased TFEB levels by 20 % (Fig. 3e). As in HeLa cells, TFEB nuclear localization and cellular concentration increased transiently in response to Torin1 and, after approximately 1 hour, decreased gradually. Of note, in MCF7 cells, at 15 hours of Torin1 treatment TFEB concentration decreased below the initial values.

Taken together, ISX-based analysis of mean population responses support Western blot findings, wherein mTOR inhibition by Torin1 increases TFEB protein levels in HeLa (Figs. 2e and 3c) as well as in MCF7 cells (Fig. 3e). Conversely, addition of fresh FM, to mildly increase mTOR activity (Fig. 1d), led to a slight, but significant, reduction in TFEB levels (Figs. 2b and 3c, e). Furthermore, these results indicate that changes in TFEB protein levels correlate with significant shifts of TFEB between nuclear and cytoplasmic compartments.

### Agglomerative clustering analysis of single cell multispectral imaging cytometry data identifies underlying subpopulation dynamics and elucidates temporal TFEB regulation

As Western blot and population-averaged ISX analyses report bulk population dynamics of TFEB, we hypothesized that cell-to-cell heterogeneity in TFEB signaling may contribute to time point variability for both approaches, and thereby contain relevant information on TFEB dynamics. Therefore, we sought to quantify subpopulation TFEB responses from single cell multispectral imaging cytometry data using SPADE-based agglomerative clustering [15].

#### Analytical framework for subpopulation analysis of TFEB distribution in time course datasets

**Fig. 4:**
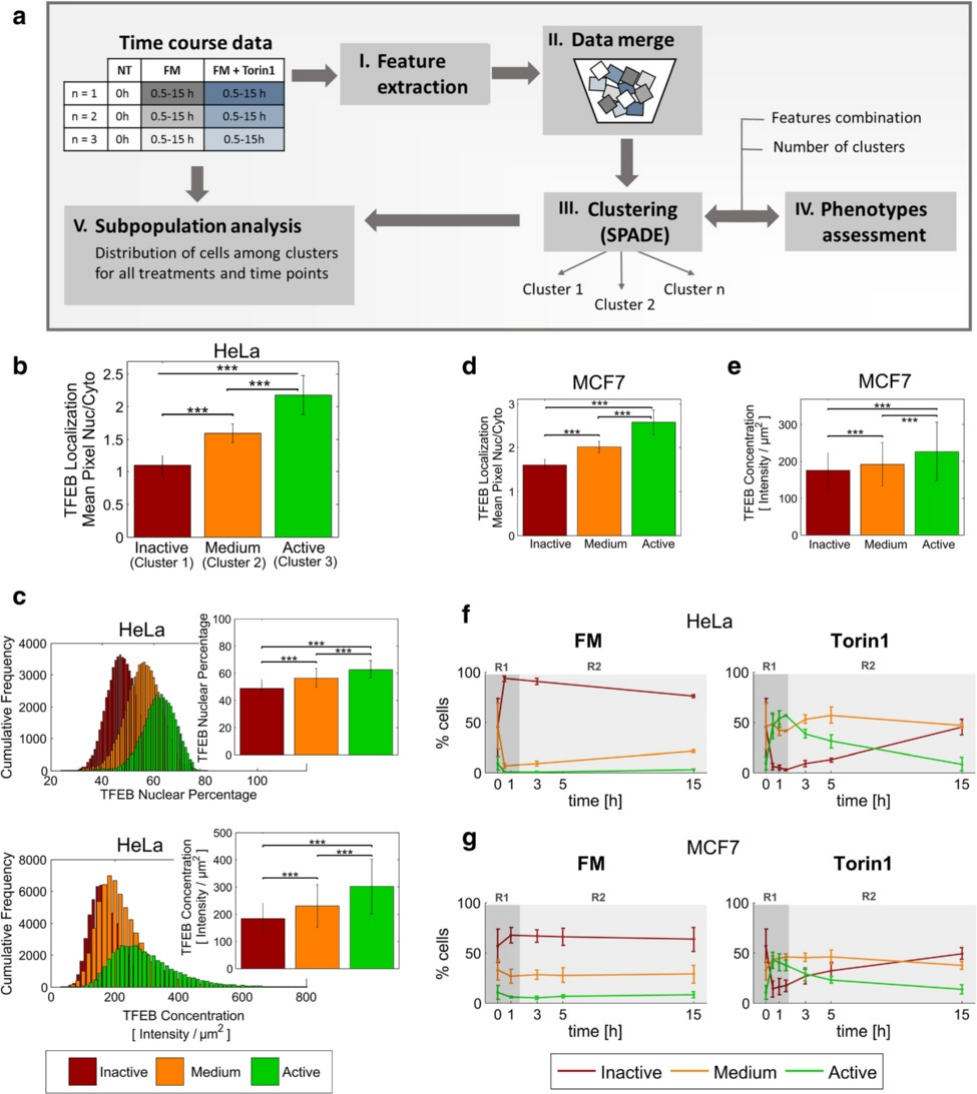
Clustering-based analysis of subpopulation dynamics of TFEB activity in response to mTOR activity modulation by fresh FM and Torin1. Subpopulation analysis of ISX multispectral imaging cytometry datasets from Fig. 3. **a** Schematic representation of the analysis workflow. Extracted feature values from all treatments and time points were merged and analyzed using SPADE software for the identification of phenotypically similar clusters. Clustering results were iteratively checked until finding a combination of features and number of clusters that yielded biologically sound and reproducible results. Finally, evolution of subpopulations was observed by tracing the percentage of cells belonging to each cluster for each treatment and time point. **b** Cluster phenotypes in HeLa cells. Bars represent the mean among all cells in each cluster ± SD (including FM, Torin1, and all repetitions and time points). The number of cells equals 48774, 42994 and 23793 for cluster 1, 2 and 3, respectively. Statistical significances were tested between clusters on 1000 randomly selected cells (two-sided Wilcoxon-rank-sum test; ***, *p* ≤ 0.001). **c** Cumulative frequency distribution for selected features that were excluded during the generation of the clusters. Bars on the top right corners display the mean value among all cells in each cluster ± SD. Statistical significances were tested between clusters on 1000 randomly selected cells (Student’s two-tailed t-test; ***, *p* ≤ 0.001). **d** Clusters phenotype in MCF7 cells. Bars represent the mean among all cells in each cluster ± SD. Statistical significances were tested between clusters on 1000 randomly selected cells (two-sided Wilcoxon-rank-sum test; ***, *p* ≤ 0.001). **e** Mean TFEB protein levels for the three clusters. Bars represent the mean among all cells in each cluster ± SD. Statistical significances were tested between clusters on 1000 randomly selected cells (Student’s two-tailed t-test; ***, *p* ≤ 0.001). **f**-**g** Subpopulation dynamics for the indicated treatments and cell lines. Reported values represent the mean among three independent experiments ± SD. Regions shaded in grey highlight different stages in TFEB dynamic response. R1: short-term rheostatic response, R2: long-term response

Our framework for subpopulation identification consists of five main steps: (I) feature extraction, (II) data merge, (III) clustering, (IV) phenotypes assessment, and (V) time course distribution analysis (Fig. 4a). In the first step, multiple quantitative features, including subcellular localization and total protein levels, are calculated for each cell from all treatments and time points. In the second step, the extracted features from all conditions are merged together, to ensure that clustering is not influenced by time points and treatments, and thus is unbiased. In the third step, the clustering algorithm SPADE is used to split the cells into a given number of groups (clusters), which should represent different phenotypes. In the fourth step, the clustering outcome is evaluated based on several criteria to assess its biological soundness, and the clustering step is iteratively repeated to establish a combination of features and cluster number (if clusters exist) for which the results satisfy all evaluation criteria. Finally, in the fifth step, we trace back the dynamic distribution of the population among the obtained clusters. Specifically, for each treatment and time point, we determine the percentage of cells belonging to each cluster. Assuming that the clusters represent biologically-meaningful phenotypes, the redistribution of cells among the different clusters should then indicate the development of subpopulations.

For this analysis to be valid, we utilize the dynamics of the subpopulation response to evaluate the consistency, i.e. biological soundness, of the clustering results (step IV) based on the following criteria:

- Criterion 1: The temporal evolution of the percentage of cells in each cluster should be consistent among repetitions, to assure the reproducibility of subpopulation dynamics. (See Additional file 4: Figure S4 and Additional file 5: Figure S5a).
- Criterion 2: The distribution of cells in each cluster should follow independent dynamics. We assume that if the distribution of cells in two or more clusters are affected in the same way by the treatments, this would indicate that the clusters are redundant (See Additional file 5: Figure S5b).

#### Identification of three TFEB activation phenotypes/subpopulations

We applied this framework to identify subpopulations in the response to FM and Torin1 treatments. The features used for this analysis included the nuclear/cytoplasmic ratio, and areas, concentrations and total intensities in the segmented compartments (cellular, nuclear and cytoplasmic masks).

After evaluating different feature combinations and number of clusters, we determined that our evaluation criteria were satisfied by the single feature “Mean Pixel Nuc/Cyto”, i.e. nuclear/cytoplasmic ratios, and a total of three, statistically different clusters. Based on the nuclear/cytoplasmic ratios, the three clusters were classified as “Inactive”, moderately active (denoted as “Medium”), and “Active”. The “Active” cluster has the highest nuclear localization and the “Inactive” cluster has the lowest nuclear localization, i.e. highest cytoplasmic retention (Fig. 4b).

We characterized the predicted clusters based on the frequency distribution and mean values of a subset of features that were not included in the cluster generation. TFEB nuclear percentage and cellular concentration features (described in Methods) yielded normal distributions within predicted clusters, with statistically-different means (Fig. 4c), further indicating that the predicted clusters represent biologically-meaningful subpopulations. Interestingly, the “Active” cluster contained the highest total cellular TFEB concentration. We confirmed this positive correlation between TFEB nuclear localization and TFEB protein levels through statistical analysis, with a correlation coefficient of 0.53 (See Additional file 1: Figure S1, panel II). To test whether TFEB protein levels and localization were correlated independently of mTOR, we increased cellular TFEB concentration by ectopic expression of GFP-TFEB, and quantified the percentage of activated cells (mainly nuclear TFEB) by fluorescence microscopy (Additional file 6: Figure S6). The amount of activated cells was significantly increased by GFP-TFEB expression compared to endogenous TFEB levels. Furthermore, the effect of overexpression was partially reversed by enhanced sequestration of TFEB in the cytosol through overexpression of 14-3-3 isoform ɣ (YWHAG), for which TFEB has a high binding affinity [6]. These results suggest that increased cellular TFEB protein levels can trigger nuclear localization and override regulation by mTOR, and that this effect is partially dependent on 14-3-3 protein levels.

We identically applied our analysis framework to define TFEB subpopulations in the MCF7 cells time course data (the workflow for defining cell line-specific subpopulations is represented in Additional file 1: Figure S1). Similar to HeLa cells, statistically-different clusters were obtained, and the cluster with the highest nuclear localization (“Active”) had the highest TFEB concentration, while the “Inactive” cluster displayed the lowest TFEB levels (Fig. 4d, e).

#### Time course subpopulation responses to FM and Torin1 treatments

The temporal impact of fresh FM and Torin1 on the population distribution among predicted activity states was calculated for HeLa cells (Fig. 4f). Results from three independent experiments are shown in Additional file 4: Figure S4. At time point 0, cells were equally distributed between the “Inactive” (45 %) and “Medium” (46 %) subpopulations, while the fraction of “Active” cells amounted to only 9 % (Fig. 4f). Thus, under basal metabolism TFEB activity is inactive-to-moderately active for most cells. In response to fresh FM, at 0.5 hours the “Inactive” subpopulation fraction increased from 45 % to nearly 100 % of the cell population, concurrent with a decrease in the “Medium” subpopulation (from 46 to 6 %) and a depletion of the “Active” subpopulation (region R1). Beginning at 1 hour, the “Inactive” subpopulation decreased, concomitant with an increased “Medium” subpopulation. At 15 hours a distribution of 21 %, 76 % and 3 % for the “Inactive”, “Medium” and “Active” subpopulations was reached, respectively (region R2). The decrease of the “Inactive” subpopulation is consistent with the reduction in mTOR activity detected 5 and 15 hours post FM treatment (Fig. 2c).

In contrast, Torin1 treatment induced a differential subpopulation response, and did not drive 100 % of the population towards a single activation state (Fig. 4f). Within 1.5 hours Torin1 reduced the “Inactive” subpopulation to marginal levels (from 45 to 2 %), slightly reduced the “Medium” subpopulation (from 46 to 41 %), and increased the “Active” subpopulation from 9 to 57 % (region R1). Following 1.5 hours, the “Inactive” and “Medium” subpopulations increased, concurrent to a decrease in “Active” subpopulation, and by 15 hours all subpopulations were similarly re-distributed to time point 0 levels, with 47 %, 45 % and 8 % of the cells in the “Inactive”, “Medium” and “Active” subpopulations, respectively (region R2).

Similar, but blunted, subpopulation redistributions were observed in MCF7 cells (Fig. 4g). FM increased the percentage of cells in the “Inactive” subpopulation (from 57 to 67 %), concomitantly decreasing the “Medium” (from 33 to 26 %) and “Active” subpopulations (from 11 to 6 %), while Torin1 decreased the “Inactive” fraction (to 14 %) and increased the “Active” (to 42 %) and “Medium” subpopulations (to 44 %). As observed in HeLa cells, TFEB activation was gradually reversed starting 1.5 hours after Torin1 treatment, nearly reaching the initial subpopulation distribution after 15 hours (region R2). Notably, differing from HeLa cell results, FM treatment did not inactivate 100 % of the population, suggesting that TFEB activity is less sensitive to mTOR activation in MCF7 cells. This is in accordance with the recently described lower mTOR control over TFEB in pancreatic cancer cells [11].

Importantly, the mean population response of the nuclear/cytoplasmic ratio could be accurately predicted based on the subpopulation distributions (see Additional file 7: Figure S7), confirming the consistency of our analysis at all time points.

Overall, the results from HeLa and MCF7 cells show that subpopulation analysis reveals highly accurate cell type-, condition-, and time-dependent phenotype dynamics. The above findings reveal that mTOR maximally induced cytoplasmic TFEB retention in all HeLa cells, but fractionally in MCF7 cells. Conversely in both cell types, Torin1 induced maximal TFEB nuclear concentration only in a fraction of cells, and following 1.5 hours of treatment, TFEB began a re-localization to the cytoplasm, suggesting an early rheostat control by mTOR followed by TFEB re-inactivation uncoupled from mTOR activity.

### Time course quantification of TFEB mean population and subpopulation responses to nutrient deprivation

**Fig. 5:**
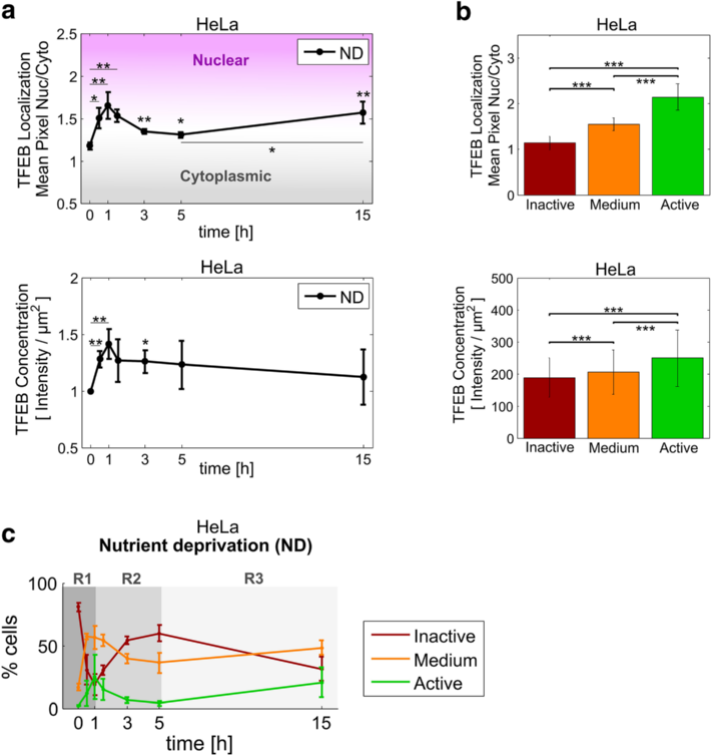
Mean population and subpopulation response of TFEB to nutrient deprivation. HeLa cells were kept in culture medium (NT, non-treated) or subjected to nutrient deprivation. At the indicated time points, cells were immunostained for TFEB and LAMP1, and Nuclei were labelled with Hoechst 33342. Following, cells were analyzed by ISX multispectral imaging cytometry. **a** Time course of the mean population response of TFEB subcellular localization and protein levels. Concentrations are shown relative to time point ‘0’. Reported values represent the mean among three independent experiments ± SD. Unless specified by horizontal lines, statistical significance was tested vs. time point ‘0’, which corresponds to the NT control (Student’s two-tailed t-test; *, *p* ≤ 0.05; **, *p* ≤ 0.01). **b** Mean TFEB subcellular localization and protein levels for the three activation phenotypes in a cell population subjected to nutrient deprivation. Bars represent the mean among all cells in each cluster ± SD (from all repetitions and time points). Statistical significance was tested between clusters on 1000 randomly selected cells (two-sided Wilcoxon-rank-sum test; ***, *p* ≤ 0.001). **c** Dynamics of the distribution of cells among the three activation phenotypes in a cell population subjected to nutrient deprivation. Reported values represent the mean among three independent experiments ± SD. Shaded regions highlight different stages in TFEB dynamic response. R1: first activation wave, R2: re-inactivation, R3: second activation wave

We then investigated a physiological perturbation on mTOR and TFEB, by subjecting HeLa cells to nutrient deprivation [6, 7]. First, we calculated the mean population responses for TFEB subcellular localization and concentration (Fig. 5a). Nutrient deprivation transiently increased the TFEB nuclear/cytoplasmic ratio (from 1.2 to maximally 1.7), peaking at 1 hour, subsequently decreasing up to 5 hours (to 1.3), and then increasing significantly again at 15 hours (to 1.6). Consistent with the findings for Torin1, concomitant to inducing nuclear translocation, nutrient deprivation induced a significant increase in the cellular concentration of TFEB, peaking at 1 hour with an increase of 41 %, and then decreasing gradually. Of note, while nuclear localization was increased again at 15 hours, TFEB protein levels stayed low.

Next, we evaluated the TFEB subpopulation response to nutrient deprivation. Cells were classified into “Active”, “Medium” and “Inactive” phenotypes using the classification model obtained above for FM and Torin1 treatments (see Additional file 1: Figure S1 for a schematic of the classification workflow). Consistent with FM and Torin1, the mean features obtained from the nutrient deprivation data set showed that the “Active” cluster, displayed the highest TFEB concentration, while the “Inactive” cluster contained the lowest TFEB levels (Fig. 5b).

Subsequently, we calculated the temporal impact of nutrient deprivation on the distribution of TFEB subpopulations (Fig. 5c). Within 1 hour, nutrient deprivation induced an initial activation wave (region R1), which reduced the “Inactive” subpopulation (from 80 to 18 %) and increased the “Medium” and “Active” subpopulations from 17 % and 3 % to 56 % and 25 %, respectively. Following 1 hour, TFEB was re-inactivated, moving 60 % of the cells to the “Inactive” phenotype after 5 hours, and leading to full loss of the “Active” subpopulation (region R2). Finally, between 5 and 15 hours TFEB subpopulation dynamics shifted again towards increasing “Medium” and “Active” phenotypes (region R3).

Thus, similar to Torin1 treatment, nutrient deprivation rapidly activated TFEB, increasing its nuclear localization. However, in contrast to Torin1 treatment, the subsequent reduction to TFEB nuclear localization was more rapid, and then reversed.

### Single cell correlation of TFEB activity states, LAMP1 concentration and lysosomal positioning in response to nutrient deprivation

**Fig. 6:**
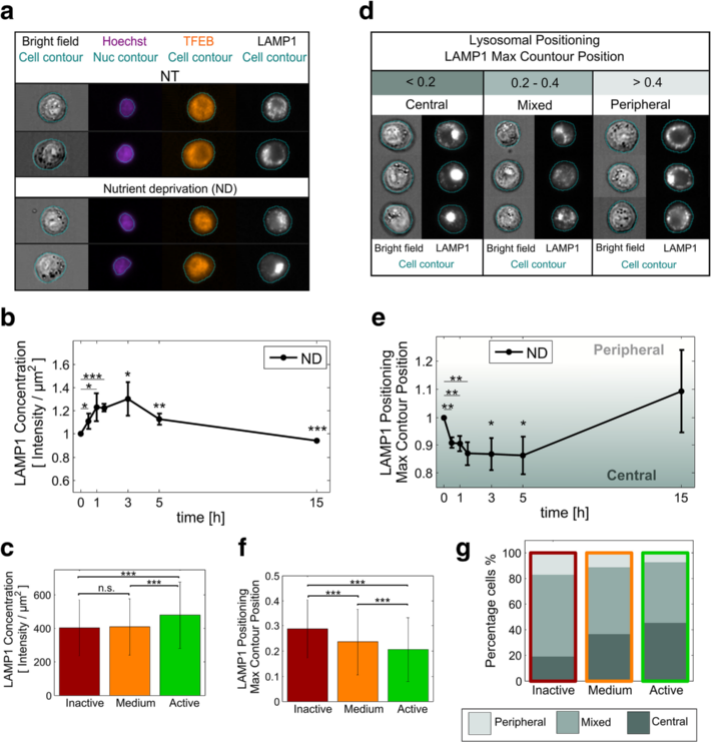
Single cell correlation of nutrient deprivation-induced TFEB activity and downstream lysosomal response by multispectral imaging cytometry. HeLa cells were kept in culture medium (NT, non-treated) or subjected to nutrient deprivation. At the indicated time points, cells were immunostained for TFEB and LAMP1, and nuclei labelled with Hoechst 33342. Following, cells were analyzed by ISX multispectral imaging cytometry. **a** Bright field and fluorescence images of representative cells for the indicated treatments. **b** Time course of the mean population response of LAMP1 protein levels, shown relative to time point ‘0’. Reported values represent the mean among three independent experiments ± SD. Statistical significance was tested vs. time point ‘0’, which corresponds to the NT control (Student’s two-tailed t-test; *, *p* ≤ 0.05; **, *p* ≤ 0.01; ***, *p* ≤ 0.001). **c** Mean LAMP1 concentration for the three TFEB activation phenotypes. Bars represent the mean among all cells in each cluster ± SD (from all repetitions and time points). Statistical significance was tested between clusters on 1000 randomly selected cells (Student’s two-tailed t-test; ***, *p* ≤ 0.001; n.s. *p* > 0.05). **d** Representative fluorescence images for different ranges of the feature “LAMP1 Max Contour Position” to assess lysosomal positioning. **e** Time course of the mean population response of “LAMP1 Max Contour Position”, shown relative to time point ‘0’. Reported values represent the mean among three independent experiments ± SD. Statistical significance was tested vs. time point ‘0’, which corresponds to the NT control (Student’s two-tailed t-test; *, *p* ≤ 0.05; **, *p* ≤ 0.01). **f** Mean “LAMP1 Max Contour Position” for the three TFEB activation phenotypes. Bars represent the mean among all cells in each cluster ± SD (from all repetitions and time points). Statistical significance was tested between clusters on 1000 randomly selected cells (two-sided Wilcoxon-rank-sum test; ***, *p* ≤ 0.001). **g** Percentage of cells with values of “LAMP1 Max Contour Position” within the ranges specified in (**d**), presented separately for each TFEB activation phenotype

We subsequently sought to gain insight into functional relevance of TFEB activity subpopulations during the response to nutrient deprivation. Therefore, we simultaneously monitored responses of endogenous TFEB and TFEB-controlled lysosomal marker LAMP1 [1, 2] by multispectral imaging cytometry (Fig. 6a). Nutrient deprivation significantly increased the cellular concentration of LAMP1 for all time points up to 5 hours, peaking at 3 hours with an increase of 30 % and decreasing thereafter (Fig. 6b). The delay in the peak in LAMP1 concentration relative to TFEB is consistent with TFEB-mediated lysosomal biogenesis. Notably, although at 15 hours TFEB accumulated in the nucleus again (Fig. 5a, c), no increase in TFEB (Fig. 5a) or LAMP1 (Fig. 6b) levels was observed at this time point. Importantly, LAMP1 concentration was significantly higher for the “Active” cluster (~17 % increase relative to “Medium” and “Inactive” clusters), demonstrating enhanced lysosomal content and thus higher TFEB downstream signaling for the phenotype classified as “Active” (Fig. 6c).

In addition, we analyzed the subcellular positioning of lysosomes, which is intricately linked to the cellular nutrient state [18]. To that end, we employed the “LAMP1 Max Contour Position” feature, defined as the location of the contour in the cell with the highest LAMP1 intensity concentration [16]. As shown in Fig. 6d and Additional file 8: Figure S8, values close to 0 indicate a centered (perinuclear) lysosomal distribution, while values close to 1 indicate a peripheral (cytoplasmic) lysosomal distribution. The mean population response (Fig. 6e) demonstrates that within 0.5 hours, as expected [18], nutrient deprivation significantly redistributed lysosomes to the nuclear region. This perinuclear lysosomal distribution was maintained up to 5 hours, and after 15 hours lysosomes re-localized towards the cell periphery. Notably, the three clusters displayed statistically different mean “LAMP1 Max Contour Position” values (Fig. 6f) and clear differences between lysosomal positioning distributions (Fig. 6g), indicating that cells in the “Inactive” cluster contain the most peripheral lysosomes and cells in the “Active” cluster contain the most perinuclear lysosomes. Consistent with the inter-cluster differences, “LAMP1 Max Contour Position” negatively correlated with TFEB nuclear/cytoplasmic ratio in single cells, with a correlation coefficient of −0.25 (see Additional file 1: Figure S1, panel III). Overall, LAMP1 concentration dynamics and spatial distribution within subpopulations indicate that the TFEB-defined subpopulations are of physiological relevance for the cellular lysosomal state.

### Maximal activation of TFEB under conditions of mTOR inhibition is negatively regulated by the proteasome, independently of TFEB concentration

#### ERK signaling does not impact TFEB activation in response to Torin1 or nutrient deprivation

**Fig. 7:**
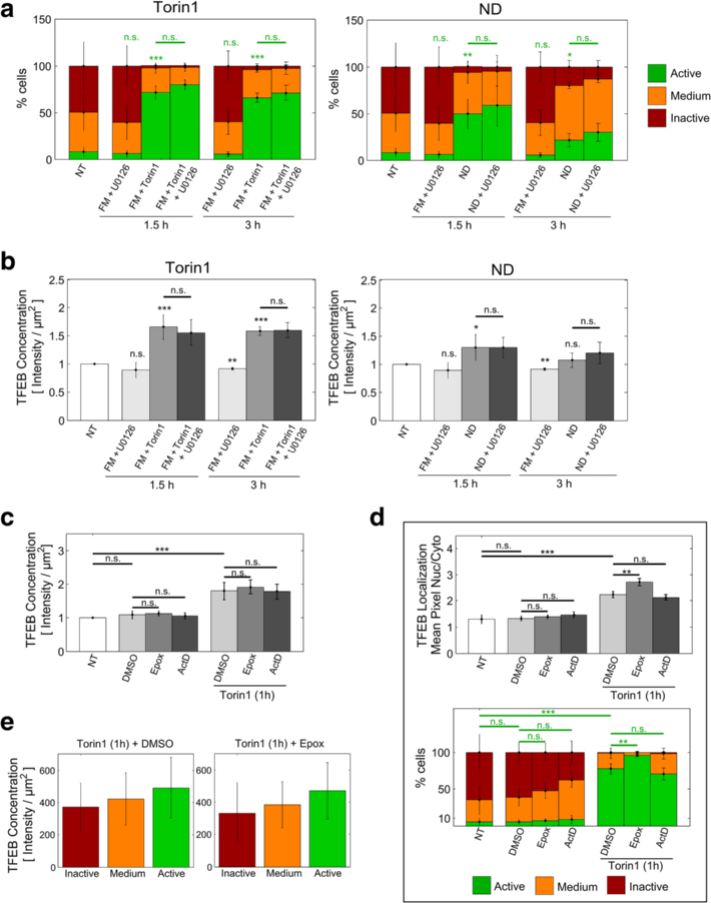
Effect of ERK, proteasome and transcriptional inhibition on mTOR inhibition-mediated TFEB activation. **a** HeLa cells were treated with fresh FM supplemented with U0126 (10 μM) alone or in co-treatment with Torin1 (2 μM), or subjected to nutrient deprivation (ND) alone or in combination with U0126. Following immunostaining for TFEB and labeling of nuclei with Hoechst 33342 cells were analyzed by multispectral imaging cytometry. For visualization, the data for FM supplemented with U0126 is included both for Torin1 (left) and for ND (right) treatments. Bars report mean subpopulation distributions among three independent experiments ± SD. Statistical significance was tested based on the “Active” subpopulation vs. non-treated control (NT), unless otherwise indicated by horizontal lines (Student’s two-tailed t-test; *, *p* ≤ 0.05; **, *p* ≤ 0.01; ***, *p* ≤ 0.001; n.s., *p* > 0.05). **b** Mean population response of TFEB concentration in HeLa cells treated as in **(a)**, shown relative to the non-treated levels (NT). Unless indicated by horizontal lines, statistical significance was tested vs. non-treated control, NT (Student’s two-tailed t-test; *, *p* ≤ 0.05; **, *p* ≤ 0.01; ***, *p* ≤ 0.001; n.s., *p* > 0.05). **c** Mean population response of TFEB concentration (relative to the non-treated levels, NT) in HeLa cells treated with Epox (1 μM), ActD (1 μg/mL) or vehicle control (DMSO) alone or in combination with Torin1 (2 μM). Bars report the mean among four independent experiments ± SD. Statistical significances were tested with Student’s two-tailed t-test (*, *p* ≤ 0.05; **, *p* ≤ 0.01; ***, *p* ≤ 0.001; n.s., non-significant). **d** Mean population response of TFEB subcellular localization and subpopulation distributions for HeLa cells treated as in (**c**). Bars report the mean among four independent experiments ± SD. Statistical significances were tested with Student’s two-tailed t-test (**, *p* ≤ 0.01; ***, *p* ≤ 0.001; n.s., non-significant). Subpopulations were compared based on the “Active” phenotype. **e** Mean TFEB concentration for the three activation phenotypes in a subset of cells treated with Torin1 alone or in co-treatment with epoxomicin, as indicated in (**c**). Bars represent the mean among all cells in each cluster for the indicated treatments ± SD

Finally, we sought to identify mechanisms which prohibit maximal TFEB activation. We first explored a possible contribution of ERK kinases, which were previously shown to negatively regulate TFEB [4]. We treated HeLa cells with the MEK1 and MEK2 inhibitor U0126 [4, 9] alone or in co-treatment with Torin1 or nutrient deprivation for 1.5 and 3 hours. We found that U0126 did not significantly impact Torin1- or nutrient deprivation-induced increases in the fraction of “Active” cells (Fig. 7a), nor in mean TFEB concentrations (Fig. 7b). Of note, treatment with fresh FM containing U0126 (Fig. 7a) led to a lesser deactivation of TFEB than treatment with fresh FM alone (Fig. 4f). Together, these results indicate that ERK signalling does not restrain the early phase of TFEB activation under the conditions tested here.

#### Maximal TFEB activation by Torin1 is negatively regulated by the proteasome

As increased TFEB activation correlated with increased TFEB protein levels (see Additional file 1: Figure S1, panel II), we next sought to determine whether the initial changes in TFEB protein levels could be attributed to proteasomal degradation or protein synthesis. Therefore, we treated HeLa cells with the proteasome inhibitor epoxomicin (1 μM) or the transcriptional inhibitor actinomycin D (1 μg/mL), alone or in combination with Torin1 for 1 hour (Fig. 7c). Neither epoxomicin nor actinomycin D had a significant effect on TFEB levels for all tested conditions, indicating that, within the period of rapid TFEB concentration changes, proteasomal degradation and transcriptional regulation do not play a significant role. We thus speculate that TFEB changes might be regulated via lysosomal degradation. However, as impairment of lysosomal function activates TFEB [9], an unbiased assessment of the involvement of lysosomal degradation on TFEB levels is not readily possible and requires future investigation.

Of note, while epoxomicin had no impact on the nuclear/cytoplasmic ratio under control conditions, nuclear TFEB localization was significantly increased under epoxomicin and Torin1 co-treatment (Fig. 7d). Subpopulation analysis demonstrates that this effect was due to a significant increase in the subpopulation with “Active” TFEB (to 96 %) (Fig. 7d), and was not related to changes in total TFEB levels (Fig. 7e).

## Discussion

**Fig. 8:**
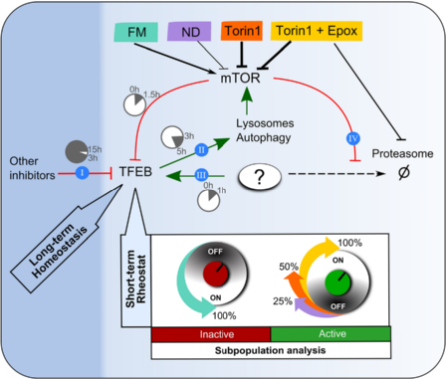
Proposed dynamic regulatory network of TFEB. TFEB is under long-term (dark blue area) and short-term (light blue area) control, regulating the strength and time span of its activation, i.e., nuclear localization. The long-term control limits the duration of TFEB activation upon sustained mTOR inhibition, displaying prominent mTOR-independent negative regulation of TFEB. The short-term control acts as a rheostat, which is highly sensitive to inactivation by mTOR, but tightly controls TFEB activation by multiple mechanisms, directly or indirectly regulated by mTOR.  Under conditions of sustained mTOR inhibition (Fig. 2d, f), starting at 3 hours, TFEB is gradually inactivated (Fig. 4f, region R2), evidencing the action of other inhibitors with slower kinetics than mTOR.  In contrast to Torin1 mTOR inhibition, nutrient deprivation displays a fast reactivation kinetics (Fig. 5c, region R2) followed by a second activation wave (Fig. 5c, region R3), suggesting that TFEB rheostat is sensitive to autophagy feedback on mTOR activity via nutrient recycling.  Proteasome inhibition by epoxomicin enhances TFEB activation (Fig. 7d), suggesting that the proteasome mediates the degradation of a “positive regulator” of TFEB, labeled here with a question mark.  The effect of epoxomicin on TFEB activation is only detectable under conditions of mTOR inhibition (Fig. 7d), suggesting that the “positive regulator” is not degraded under conditions of high mTOR activity (i.e., mTOR activity inhibits the degradation of the “positive regulator”)

Here we investigated the relationship between modulations to mTOR activity and the consequent changes to localization and concentration of TFEB in HeLa and MCF7 cells. We report the novel findings that mTOR exerts a rapid, time-limited rheostat control on TFEB subcellular localization and protein levels (see Fig. 8). During the period of 0.5 to 1.5 hours following perturbation, mTOR activation decreased TFEB protein levels and increased TFEB cytoplasmic retention. Conversely, in response to mTOR inhibition by either Torin1 or nutrient deprivation, during this period, TFEB protein levels rapidly increased concurrent to enhanced accumulation of TFEB in the nucleus. Surprisingly, this effect was limited in duration to a period of 3 hours, and during the period of 3 to 15 hours, TFEB concentrations and subcellular distributions returned towards basal levels for all conditions, evidencing homeostatic regulatory mechanisms dependent and independent of mTOR. While we show that mTOR activation maximally inhibits TFEB, mTOR inhibition only fractionally activated TFEB. Furthermore, our findings indicate that ERK signaling exerts a negligible inhibitory effect on TFEB under nutrient deprivation and Torin1 conditions, and instead indicate a role for proteasome degradation pathways in the regulation of TFEB subcellular localization (see Fig. 8) independent of TFEB levels.

### Subpopulation analysis reveals accurate TFEB signaling behavior

We achieved highly accurate analysis of spatial and temporal TFEB dynamics by subpopulation analysis. To decompose cellular heterogeneity into discrete subpopulations of TFEB activation states we used agglomerative clustering [19–21] of single-cell imaging datasets, and established criteria to optimize the number of phenotypes and input features. We identified “Active”, “Medium” and “Inactive” TFEB subpopulations which significantly and reproducibly correlated with TFEB signaling. Consistent with TFEB activation of lysosomal biogenesis [4], the “Active” subpopulation reported higher lysosomal content than “Medium” and “Inactive” subpopulations (Fig. 6c). Moreover, “Active”, “Medium” and “Inactive” subpopulations reported significant differences in lysosomal positioning (Fig. 6f, g), an indicator of the cellular metabolic state. In response to nutrient deprivation, the “Active” subpopulation was enriched with perinuclear lysosomes, which report a starvation response, while the “Inactive” was enriched with mixed and peripheral lysosomes, which indicate normal growth conditions [18]. We therefore conclude that subpopulation analysis of TFEB distribution and concentration reveals the time course response of TFEB to metabolic stress.

### mTOR activity modulations induce distinct time-evolving TFEB subpopulation redistributions

Quantifying TFEB subpopulation redistributions over time, we found that with the addition of fresh FM, which mildly increased mTOR activity (Fig. 1d), TFEB heterogeneity was rapidly lost in HeLa cells (Fig. 4f). In contrast, consistent but smaller population shifts were measured in MCF7 cells (Fig. 4g), suggesting a cell line-dependent lower sensitivity to mTOR activation, possibly related to altered nuclear import of TFEB [11]. Notably, subpopulation analysis revealed that at 15 hours post FM treatment, a small percentage of HeLa cells switched to the less active “Medium” phenotype. This subpopulation shift may reflect the consumption of nutrients and/or growth factors, as indicated by decreased 4E-BP1 phosphorylation (Fig. 2c).

In contrast to FM, in both HeLa and MCF7 cells, Torin1 rapidly depleted the “Inactive” subpopulation and increased “Active” and “Medium” subpopulations (Fig. 4f, g). Strikingly, while Torin1 inhibition of mTOR was maintained for 15 hours (Fig. 2f), TFEB re-inactivation began 1.5 hours following Torin1 treatment and required 15 hours to inactive approximately 50 % of the population, compared to 100 % inactivation by fresh FM at 0.5 hours. These findings evidence an additional mTOR-independent, negative regulatory mechanism, engaged similarly in HeLa and MCF7 cells under conditions of prolonged mTOR inhibition. Putative mechanisms may involve phosphorylation by GSK3 [12], multiple serine phosphorylations [4, 22], and/or other post-translational modifications such as SUMOylation [23].

Notably, nutrient deprivation-mediated activation of TFEB differed from Torin1 treatment. While Torin1 strongly induced the “Active” subpopulation and depleted the “Inactive” subpopulation (Fig. 4f), nutrient deprivation mostly increased the “Medium” subpopulation without depleting the “Inactive” subpopulation (Fig. 5c). Further, distinct from Torin1 treatment, between 1.5 and 5 hours (R2 period) TFEB was rapidly inactivated, and between 5 and 15 hours (R3 period) both “Active” and “Medium” subpopulations increased, suggesting a second activation wave.

We suggest this alternating behavior was due to negative feedback of autophagy on mTOR, as we previously predicted by agent-based modeling [24]. Here we report that lysosomal content and perinuclear clustering were maximal during the first 5 hours of nutrient deprivation (Fig. 6b, e), indicative of maximal autophagy activation, which could provide recycled nutrients sufficient to re-activate mTOR and thereby inactivate TFEB. Subsequently, after 5 hours, depletion of autophagy recycled nutrients could lead to renewed inactivation of mTOR, which is in agreement with the observed re-activation of TFEB at 15 hours of nutrient deprivation. Interestingly, at this time point TFEB nuclear localization is not paralleled with increases in either TFEB or LAMP1 protein levels. Moreover, also at 15 hours, lysosomes localized at the cell periphery (Fig. 6e). These observations might be due to depletion of intracellular substrates following prolonged nutrient deprivation. Future studies will explore whether peripheral lysosomal redistribution reflects a cellular shift toward exploiting endocytic nutrient sources.

### During pharmacological mTOR inhibition the proteasome negatively regulates nuclear localization of TFEB

Notably, we show that under Torin1 treatment and during early phases of nutrient deprivation TFEB protein levels correlate with subcellular localization. This is consistent with increased transcription of autophagy and lysosomal genes following TFEB overexpression [1, 2, 6]. Using specific inhibitors, we ruled out that initial (within 1 hour) increases and decreases in TFEB levels were due to protein translation or proteasomal activities (Fig. 7c), indicating that transcriptional feedback [25] is not relevant during the first hour of treatment. These results further suggest that TFEB may be targeted for lysosomal degradation, thereby forming a negative feedback circuit. However, lysosomal stress potently activates TFEB [9] and therefore putative lysosomal targeting of TFEB requires further investigation.

Importantly, we found that mTOR inactivation failed to maximally activate TFEB in all cells of a cell population (Fig. 4f, g). Our findings exclude ERK signaling as a limiting factor (Fig. 7a). Instead, we identified a novel TFEB inhibitory role for the proteasome, under conditions of mTOR inhibition. Selectively in Torin1-treated cells, epoxomicin-mediated inhibition of the proteasome leads to “Active” nuclear localization of TFEB in nearly 100 % of cells (Fig. 7d), without altering TFEB protein levels (Fig. 7c, e). The mechanism by which the proteasome negatively regulates TFEB remains to be determined. We propose an indirect pathway, in which mTOR activity inhibits proteasomal degradation of a positive TFEB regulator. Thereby, maximal activation of TFEB nuclear translocation requires inhibition of mTOR and proteasome activities (see Fig. 8). Alternatively, proteasome inhibition might activate TFEB via induction of proteotoxic stress [26], which might be exacerbated under conditions of decreased mTOR activity [27].

## Conclusions

Overall, we demonstrate that TFEB levels and subcellular distribution undergo distinct short-term and long-term control. Our findings suggest that the rapid rheostatic response, mediated by mTOR, allows the cell to quickly adapt to metabolic changes, while the long-term, mTOR-independent homeostatic response controls the magnitude and duration of TFEB activation, and presumably limits excessive autophagy. Our findings also suggest that TFEB may be targeted by lysosomes, and that under conditions of mTOR inhibition the proteasome regulates the early response of TFEB localization, uncoupled from changes to TFEB levels. As TFEB is a central player in cancer [11, 12], our approach to time series analyses of magnitude and dynamics of subpopulation shifts enables biomarker assessment of cell line sensitivity and responsiveness. We propose our approach as a useful general framework for identifying and quantifying information contained within heterogeneous imaging datasets.

## Abbreviations

ActD, actinomycin D; Epox, epoxomicin; FM, fresh, fully-supplemented medium; ISX, ImageStreamX imaging flow cytometer; LAMP1, lysosomal-associated membrane protein 1; mTOR, mammalian target of rapamycin; ND, nutrient deprivation; NT, non-treated; SPADE, spanning-tree progression analysis of density-normalized events; TFEB, transcription factor EB; WF, wide field imaging

## Additional files

Additional file 1: Figure S1. Work flow for classification of cell subpopulations. Initially, cells subjected to FM or Torin1 treatments are classified into three groups/clusters (denoted as activation phenotypes) using agglomerative clustering on the base-clustering-feature “Mean Pixel Nuc/Cyto” (See Fig. 4a). The resulting classification criteria, consisting of thresholds on the base-clustering-feature, constitute our data-based model for cell classification. This model is estimated separately for HeLa and MCF7 cells, and thus, is cell line specific. The activation phenotypes are further characterized by identifying additional phenotypic differences between the cell groups. To this end, the three clusters are statistically compared based on a set of features which were not used in the generation of the cell classification model. Besides identifying features which are specific to each activation phenotype, significant differences between the cell groups report correlations between the evaluated features and the base clustering feature (exemplified by the correlation coefficients and correlation plots on the top right corners of the grey panels). Finally, the FM and Torin1 data-based model is used as a basis for cell classification in response to other treatments such as nutrient deprivation, and inhibition of ERK, proteasome or protein translation (model extrapolation). (JPG 7051 kb) Additional file 2: Figure S2. Comparison of imaging cytometry (ISX) and wild-field microscopy (WF) approaches to measure endogenous TFEB subcellular distribution. Representative WF and ISX immunofluorescence images of endogenous TFEB in HeLa cells treated with fresh full medium (FM, 0.5 hours), Torin1 (1 hour) or left untreated (NT). Graphs represent normalized intensity profiles for Hoechst (purple) and TFEB (orange) along the indicated red line. Both measurement approaches show similar intensity profiles for each experimental condition. (JPG 2766 kb) Additional file 3: Figure S3. Representative imaging cytometry images of endogenous TFEB under different treatments with indicated nuclear and cellular contours. (a) Non- treated: Most cells display slightly higher cytosolic concentration, and some cells display similar concentrations in nuclear and cytosolic compartments. (b) Fresh full medium: Cells display higher cytosolic than nuclear concentration. (c) Torin1: Cells display higher nuclear than cytosolic concentration. (JPG 4456 kb) Additional file 4: Figure S4. Positive clustering example. To demonstrate the reproducibility of the clustering outcome, we separately present the curves from three independent experiments (rows 1 to 3) and the combined mean response ± SEM (row 4), which corresponds to the subpopulation dynamics presented in Fig. 4f. The result was obtained using three clusters and the feature “Mean Pixel Nuc/Cyto” as input. Importantly, treatments with FM and Torin1 induced a clear redistribution of the cell population among the different phenotypes (clusters). This distribution was consistent among the three repetitions and displayed independent dynamics for each cluster, thus adhering to our first and second evaluation criteria, respectively. (JPG 1499 kb) Additional file 5: Figure S5. Negative clustering examples. (a) Example of a clustering outcome dissatisfying criterion 1, i.e., reproducibility of the dynamic distribution of cells among clusters. The result was obtained using three clusters with the following input features: area cell, concentration cell, and “Mean Pixel Nuc/Cyto”. In this case, the evolution in time of the percentage of cells in clusters 1 (blue) and 2 (black) is not reproducible. (b) Example of a clustering outcome dissatisfying criterion 2, i.e., non-redundant dynamics. The result was obtained using four clusters with the input feature “Mean Pixel Nuc/Cyto”. In this case, clusters 1 (black) and 3 (blue) follow similar dynamic responses to all treatments, indicating that the two clusters are redundant. (JPG 2839 kb) Additional file 6: Figure S6. Nuclear localization of TFEB is influenced by total levels of cellular TFEB. (a) HeLa cells were subjected or not to transfections with the indicated constructs and treated 24 hours post transfection with 2 μM Torin1 for 3 hours or left non-treated (NT). Representative images demonstrate the subcellular distribution of TFEB fluorescence for endogenous TFEB (TFEB immunofluorescence, IF), or transiently overexpressed GFP-TFEB, expressed alone or coexpressed with RFP-tagged 14-3-3 protein isoform YWHAG. The look-up-table ‘Fire’ (ImageJ) was applied to grey scale images of TFEB or GFP-TFEB fluorescence, representing ranging from high (white) to low (dark purple) intensity values, as displayed in color scale bar. Scale bars (white line), 20 μm. (b) Quantification of the number of cells with mainly nuclear TFEB fluorescence. At least 30 cells were scored per condition and experiment in three independent experiments. Statistical significance was tested using two-tailed Student’s t-test (**, *p* ≤ 0.01; ***, *p* ≤ 0.001). (JPG 1702 kb) Additional file 7: Figure S7. Mean population response of nuclear/cytoplasmic ratio predicted from subpopulation distributions. Dots and error bars: mean population response ± SD found experimentally, as presented in Fig. 3b, d. Dotted lines: mean population response predicted based on subpopulation distributions. The predicted mean population response (F) was estimated as follows  F = ( α_I_F_I _+ α_M_F_M _+ α_A_F_A_ )/100, where  F_I_, F_M_ and F_A_ are the mean “Mean Pixel Nuc/Cyto” of the “Inactive”, “Medium” and “Active” clusters, respectively, and α_I_, α_M_ and α_A_ are the percentage of cells in each cluster.  F_I_, F_M_ and F_A_ are constants (presented in Fig. 4b, d), while and α_I_, α_M_ and α_A_ are functions of time given by the curves in Fig. 4f, g. (JPG 657 kb) Additional file 8: Figure S8. Extended representative data set of lysosomal positioning. (a) Schematic representation of the feature “MAX Contour Position” used to quantify lysosomal positioning. (b) Representative LAMP1 immunofluorescence images for different ranges of the feature “LAMP1 MAX Contour Position” in HeLa cells treated as in Fig. 6. (JPG 3911 kb)
