# Supplementary figures and images for: Time course decomposition of cell heterogeneity in TFEB signaling states reveals homeostatic mechanisms restricting the magnitude and duration of TFEB responses to mTOR activity modulation
Source: BMC Cancer. 2016 Jun 7;16:355. doi: 10.1186/s12885-016-2388-9 (PMC4896000; doi:10.1186/s12885-016-2388-9)

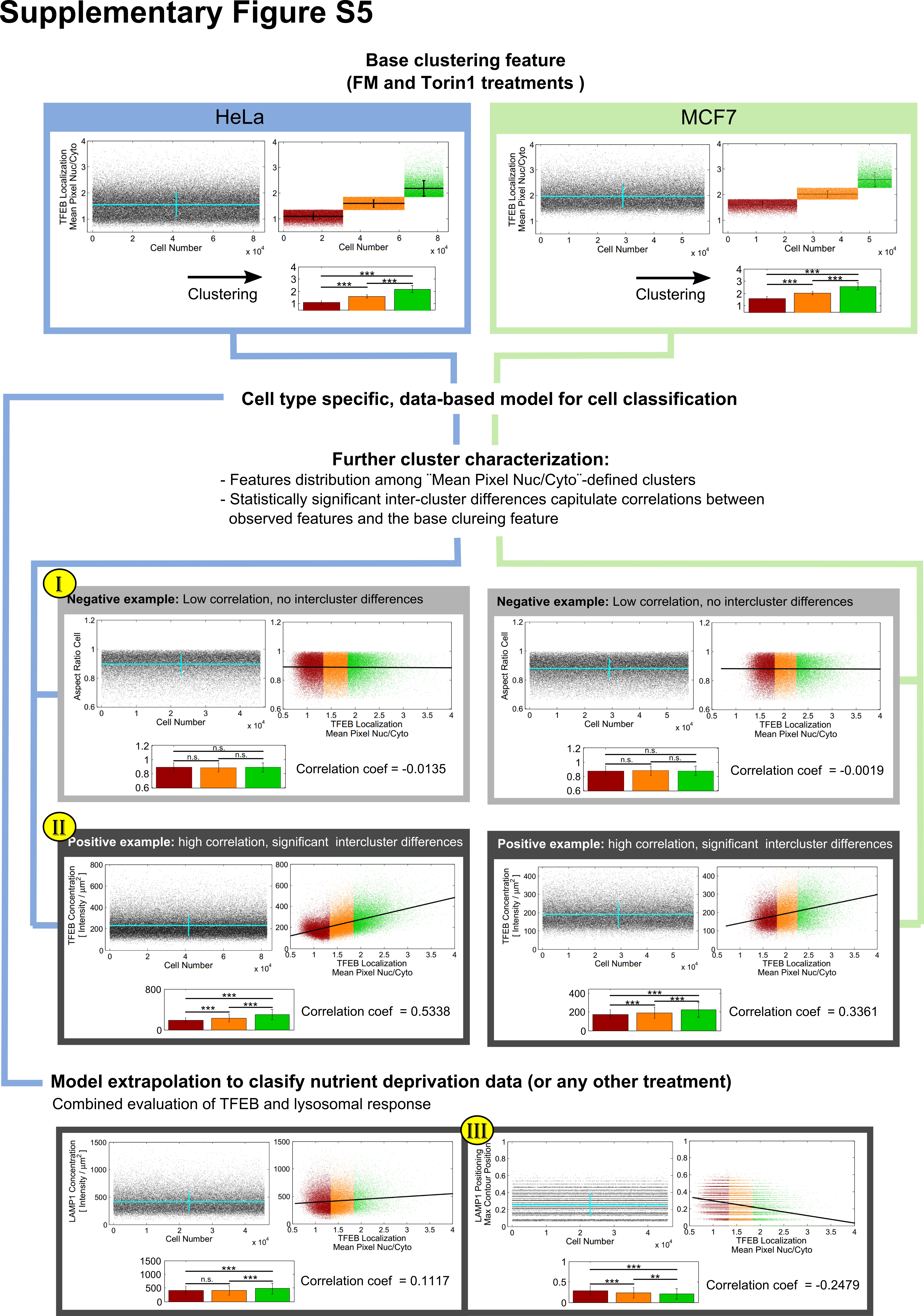

Supplement: Additional file 1: Figure S1. — Work flow for classification of cell subpopulations. Initially, cells subjected to FM or Torin1 treatments are classified into three groups/clusters (denoted as activation phenotypes) using agglomerative clustering on the base-clustering-feature “Mean Pixel Nuc/Cyto” (See Fig. 4a). The resulting classification criteria, consisting of thresholds on the base-clustering-feature, constitute our data-based model for cell classification. This model is estimated separately for HeLa and MCF7 cells, and thus, is cell line specific. The activation phenotypes are further characterized by identifying additional phenotypic differences between the cell groups. To this end, the three clusters are statistically compared based on a set of features which were not used in the generation of the cell classification model. Besides identifying features which are specific to each activation phenotype, significant differences between the cell groups report correlations between the evaluated features and the base clustering feature (exemplified by the correlation coefficients and correlation plots on the top right corners of the grey panels). Finally, the FM and Torin1 data-based model is used as a basis for cell classification in response to other treatments such as nutrient deprivation, and inhibition of ERK, proteasome or protein translation (model extrapolation). (JPG 7051 kb) [file 12885_2016_2388_MOESM1_ESM.jpg]

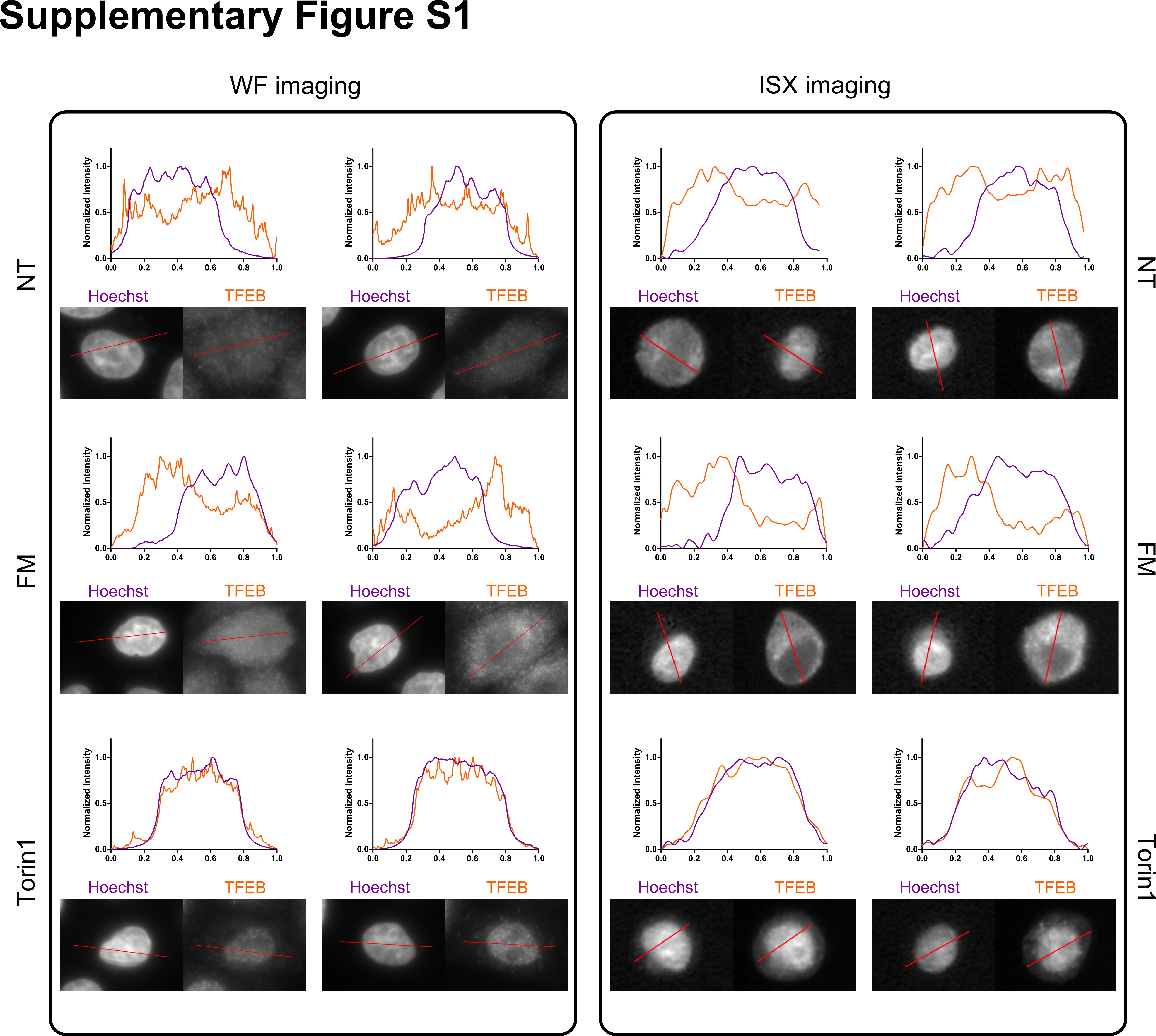

Supplement: Additional file 2: Figure S2. — Comparison of imaging cytometry (ISX) and wild-field microscopy (WF) approaches to measure endogenous TFEB subcellular distribution. Representative WF and ISX immunofluorescence images of endogenous TFEB in HeLa cells treated with fresh full medium (FM, 0.5 hours), Torin1 (1 hour) or left untreated (NT). Graphs represent normalized intensity profiles for Hoechst (purple) and TFEB (orange) along the indicated red line. Both measurement approaches show similar intensity profiles for each experimental condition. (JPG 2766 kb) [file 12885_2016_2388_MOESM2_ESM.jpg]

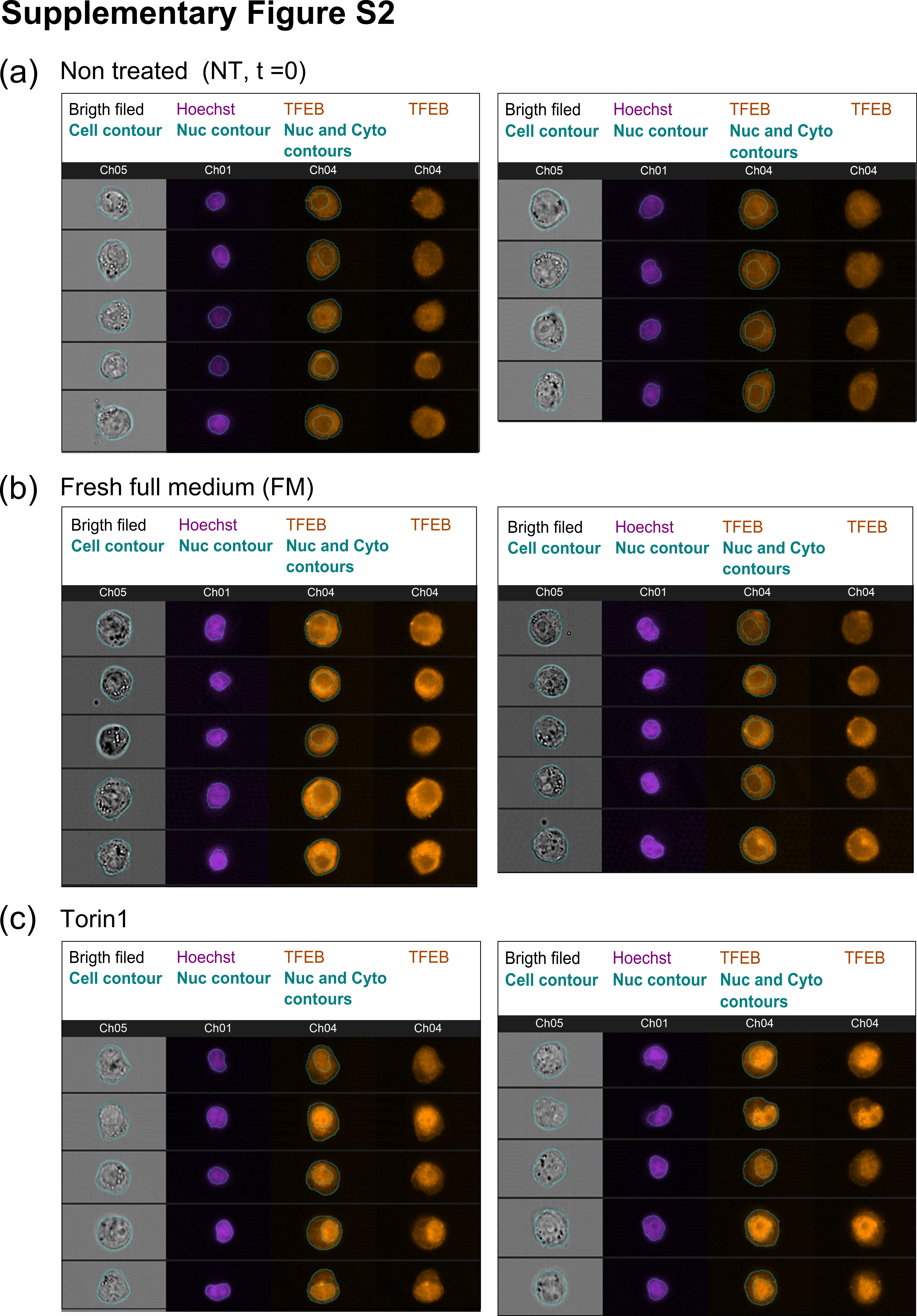

Supplement: Additional file 3: Figure S3. — Representative imaging cytometry images of endogenous TFEB under different treatments with indicated nuclear and cellular contours. (a) Non- treated: Most cells display slightly higher cytosolic concentration, and some cells display similar concentrations in nuclear and cytosolic compartments. (b) Fresh full medium: Cells display higher cytosolic than nuclear concentration. (c) Torin1: Cells display higher nuclear than cytosolic concentration. (JPG 4456 kb) [file 12885_2016_2388_MOESM3_ESM.jpg]

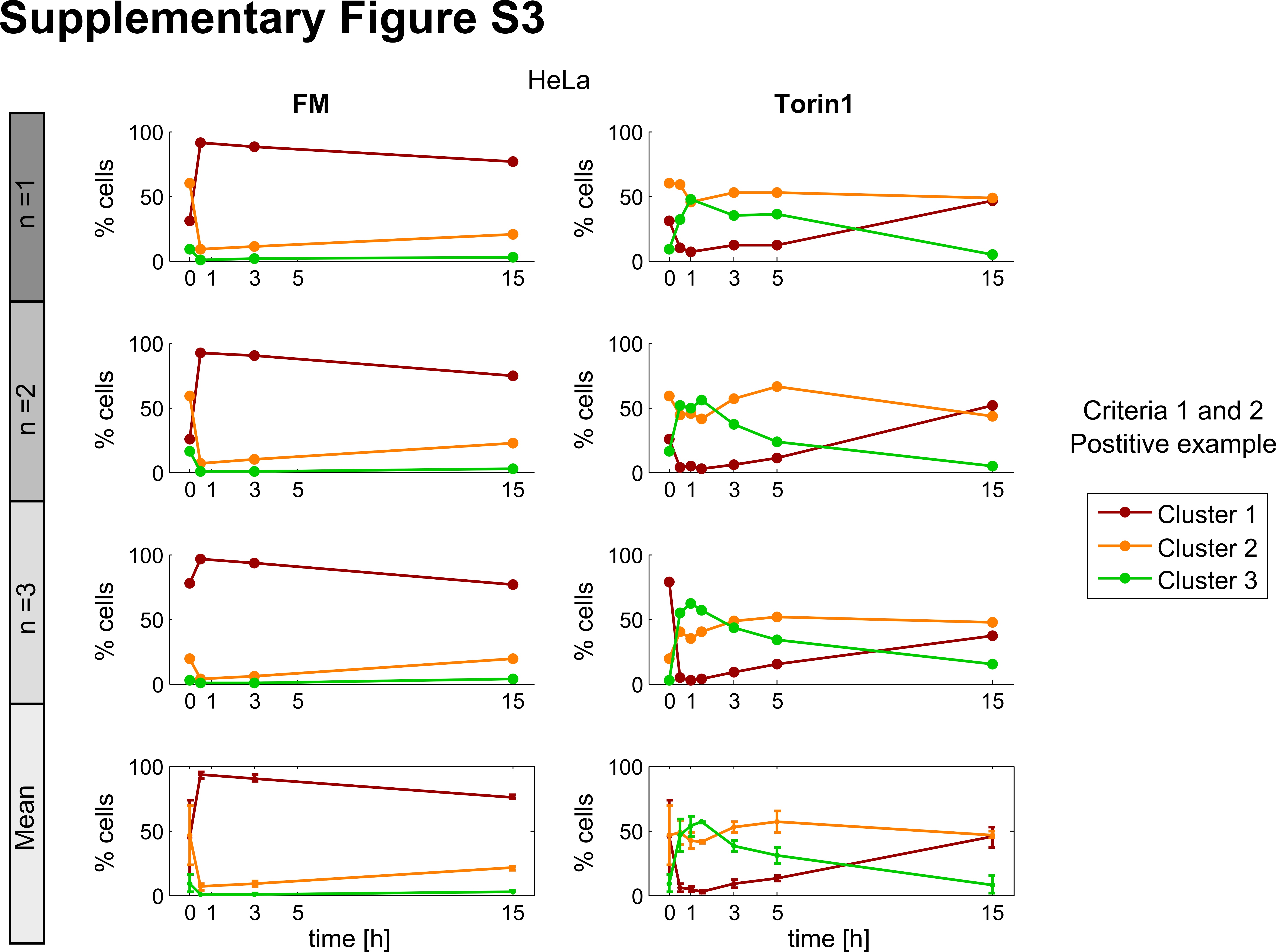

Supplement: Additional file 4: Figure S4. — Positive clustering example. To demonstrate the reproducibility of the clustering outcome, we separately present the curves from three independent experiments (rows 1 to 3) and the combined mean response ± SEM (row 4), which corresponds to the subpopulation dynamics presented in Fig. 4f. The result was obtained using three clusters and the feature “Mean Pixel Nuc/Cyto” as input. Importantly, treatments with FM and Torin1 induced a clear redistribution of the cell population among the different phenotypes (clusters). This distribution was consistent among the three repetitions and displayed independent dynamics for each cluster, thus adhering to our first and second evaluation criteria, respectively. (JPG 1499 kb) [file 12885_2016_2388_MOESM4_ESM.jpg]

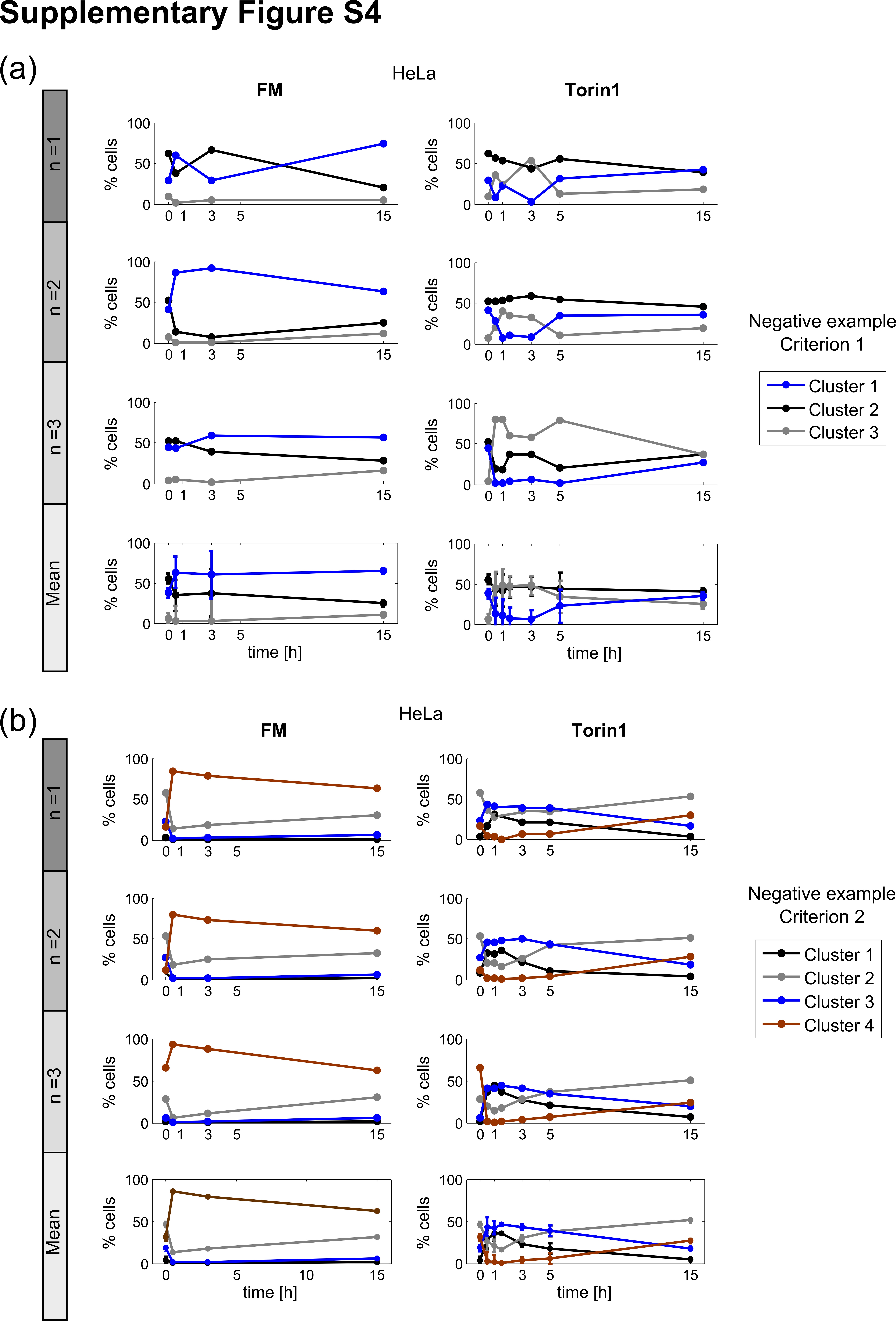

Supplement: Additional file 5: Figure S5. — Negative clustering examples. (a) Example of a clustering outcome dissatisfying criterion 1, i.e., reproducibility of the dynamic distribution of cells among clusters. The result was obtained using three clusters with the following input features: area cell, concentration cell, and “Mean Pixel Nuc/Cyto”. In this case, the evolution in time of the percentage of cells in clusters 1 (blue) and 2 (black) is not reproducible. (b) Example of a clustering outcome dissatisfying criterion 2, i.e., non-redundant dynamics. The result was obtained using four clusters with the input feature “Mean Pixel Nuc/Cyto”. In this case, clusters 1 (black) and 3 (blue) follow similar dynamic responses to all treatments, indicating that the two clusters are redundant. (JPG 2839 kb) [file 12885_2016_2388_MOESM5_ESM.jpg]

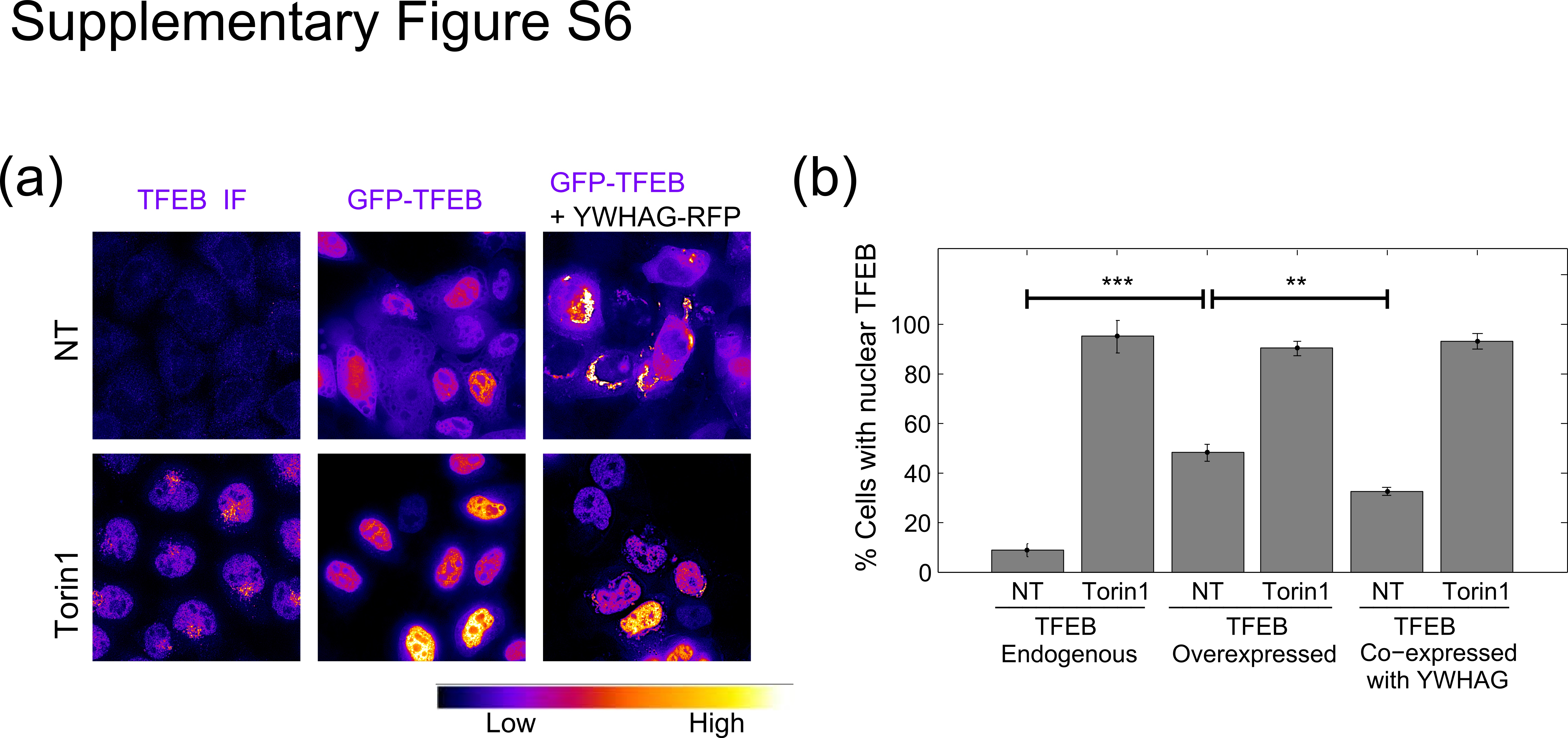

Supplement: Additional file 6: Figure S6. — Nuclear localization of TFEB is influenced by total levels of cellular TFEB. (a) HeLa cells were subjected or not to transfections with the indicated constructs and treated 24 hours post transfection with 2 μM Torin1 for 3 hours or left non-treated (NT). Representative images demonstrate the subcellular distribution of TFEB fluorescence for endogenous TFEB (TFEB immunofluorescence, IF), or transiently overexpressed GFP-TFEB, expressed alone or coexpressed with RFP-tagged 14-3-3 protein isoform YWHAG. The look-up-table ‘Fire’ (ImageJ) was applied to grey scale images of TFEB or GFP-TFEB fluorescence, representing ranging from high (white) to low (dark purple) intensity values, as displayed in color scale bar. Scale bars (white line), 20 μm. (b) Quantification of the number of cells with mainly nuclear TFEB fluorescence. At least 30 cells were scored per condition and experiment in three independent experiments. Statistical significance was tested using two-tailed Student’s t-test (**, p ≤ 0.01; ***, p ≤ 0.001). (JPG 1702 kb) [file 12885_2016_2388_MOESM6_ESM.jpg]

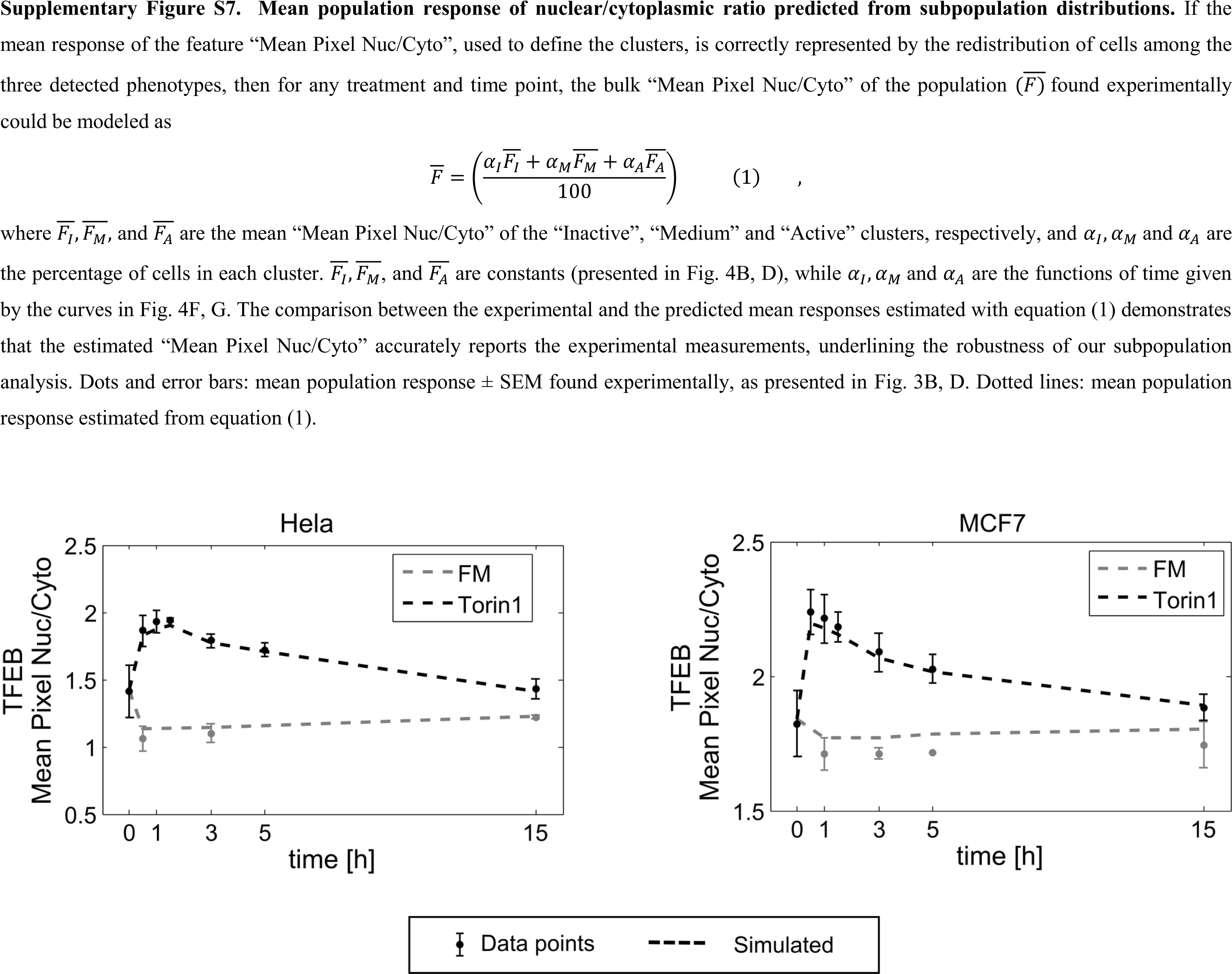

Supplement: Additional file 7: Figure S7. — Mean population response of nuclear/cytoplasmic ratio predicted from subpopulation distributions. Dots and error bars: mean population response ± SD found experimentally, as presented in Fig. 3b, d. Dotted lines: mean population response predicted based on subpopulation distributions. The predicted mean population response (F) was estimated as follows F = ( αIFI + αMFM + αAFA )/100, where FI, FM and FA are the mean “Mean Pixel Nuc/Cyto” of the “Inactive”, “Medium” and “Active” clusters, respectively, and αI, αM and αA are the percentage of cells in each cluster. FI, FM and FA are constants (presented in Fig. 4b, d), while and αI, αM and αA are functions of time given by the curves in Fig. 4f, g. (JPG 657 kb) [file 12885_2016_2388_MOESM7_ESM.jpg]

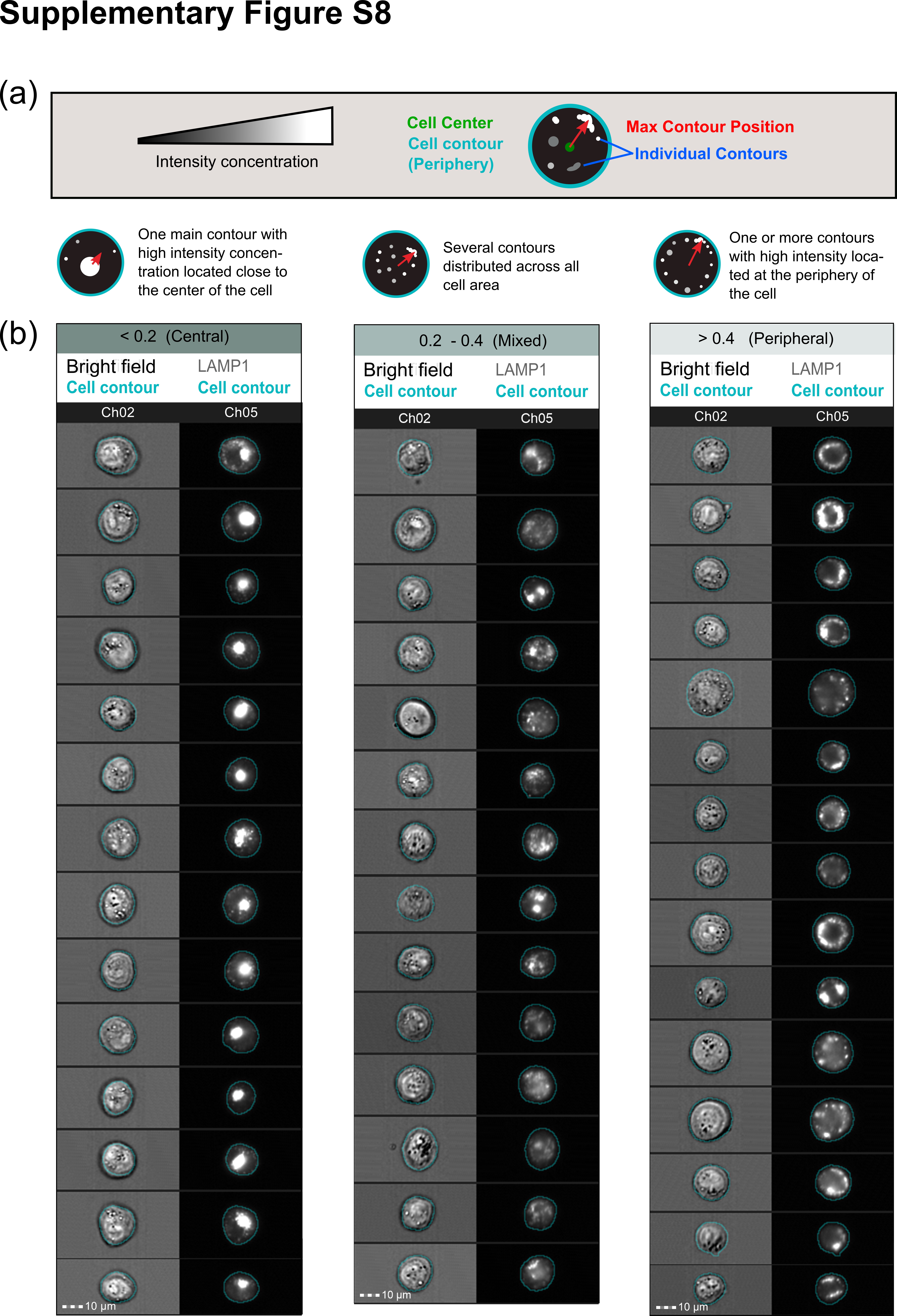

Supplement: Additional file 8: Figure S8. — Extended representative data set of lysosomal positioning. (a) Schematic representation of the feature “MAX Contour Position” used to quantify lysosomal positioning. (b) Representative LAMP1 immunofluorescence images for different ranges of the feature “LAMP1 MAX Contour Position” in HeLa cells treated as in Fig. 6. (JPG 3911 kb) [file 12885_2016_2388_MOESM8_ESM.jpg]
